# The Relationship of Need for Cognition and Typical Intellectual Engagement with Intelligence and Executive Functions: A Multi-Level Meta-Analysis

**DOI:** 10.3390/jintelligence13110142

**Published:** 2025-11-06

**Authors:** Felix M. Schweitzer, Nele M. Lindenberg, Monika Fleischhauer, Sören Enge

**Affiliations:** Institute of Psychosocial Research for Health Promotion and Intervention (IHPI), Department of Psychology, Faculty of Natural Sciences, MSB Medical School Berlin, 14197 Berlin, Germany; nele.lindenberg@student.medicalschool-berlin.de (N.M.L.); monika.fleischhauer@medicalschool-berlin.de (M.F.); soeren.enge@medicalschool-berlin.de (S.E.)

**Keywords:** need for cognition, typical intellectual engagement, intelligence, executive functions, meta-analysis

## Abstract

In this preregistered multi-level meta-analysis, we aim to clarify the association of need for cognition (NFC) and typical intellectual engagement (TIE) with intelligence and executive functions. Multi-level models with robust variance estimation were specified and risk of bias was assessed with the adapted Risk of Bias Utilized for Surveys Tool. NFC/TIE was associated with fluid intelligence (Gf; *r* = 0.18, *p* < .001, *N* = 25,367), crystallized intelligence (Gc; *r* = 0.26, *p* < .001, *N* = 14,651), general intelligence (*r* = 0.23, *p* < .001, *N* = 8479), and working memory (WM) capacity (*r* = 0.15, *p* < .001, *N* = 5921). Associations with WM updating (*r* = 0.08, *p* = .111, *N* = 1084), inhibition (*r* = 0.04, *p* = .077, *N* = 2895), and shifting (*r* = 0.01, *p* = 0.642, *N* = 1727) were non-significant. NFC (*r* = 0.19) was more strongly related to Gf than TIE (*r* = 0.12; *F*(1, 12.10) = 5.04, *p* = .045) whereas TIE (*r* = 0.35) was more strongly associated with Gc than NFC (*r* = 0.24; *F*(1, 13.10) = 10.70, *p* = .006). Correlations with Gc significantly declined over time (*b*_1_ = −0.006, β_1_ = −0.04, *p* = .010). Results provide strong evidence for small-to-moderate associations of NFC and TIE with Gf, Gc, general intelligence, and WM capacity, and at best small associations with core executive functions.

## 1. Introduction

Making sense of an increasingly complex world arguably requires not only certain cognitive abilities but also the motivation to invest effort in understanding complex topics and problems. Numerous personality traits have been conceptualized that capture different, often strongly related aspects of cognitive motivation, i.e., an individual’s tendency to actively seek out, engage in, and enjoy cognitively demanding activities, such as reading about a new topic, trying to understand a difficult problem, and developing solutions to it. One of these traits, the need for cognition (NFC; Cacioppo and Petty 1982), has sparked considerable research interest over the last few decades. While one might expect that individuals with a higher expression of this trait also possess more enhanced cognitive abilities, such as intelligence, empirical results differ considerably between studies (e.g., Tidwell et al. 2000; Stuart-Hamilton and McDonald 2001; Fleischhauer et al. 2010; Hill et al. 2013; Furnham and Thorne 2013). The purpose of this meta-analysis is to examine the relationship of NFC and the closely related typical intellectual engagement (TIE) with intelligence, focusing on fluid, crystallized, and general intelligence, as well as shifting, inhibition, and working memory. We aim to provide a detailed picture of these relations based on the currently accessible literature in a multi-level meta-analysis. Several moderators are included which may explain the heterogeneity of effects.

### 1.1. Cognitive Motivation

One of the most prominent of constructs targeting cognitive motivation is arguably NFC, conceptualized by Cacioppo and Petty (1982) and characterized as an individual’s “tendency to engage in and enjoy effortful cognitive activity” (Cacioppo et al. 1996, p. 197). Individuals scoring high on NFC have been found, for example, to be intrinsically motivated and naturally inclined to seek, acquire, and evaluate information to make sense of their surroundings (Cacioppo et al. 1996). The trait has therefore been classified as one of 34 investment traits (Von Stumm and Ackerman 2013). Over the years, other constructs such as openness for ideas (Costa and McCrae 1992), TIE (Goff and Ackerman 1992), and epistemic curiosity (Litman 2008) have been argued to be closely related to NFC. TIE, for instance, was introduced by Goff and Ackerman (1992) out of theoretical interest in the connection between personality and cognitive abilities. It has been defined as “an individual’s aversion or attraction to tasks that are intellectually taxing and is thus related to acculturative and purposeful development and expression of certain intellectual abilities” (Ackerman et al. 1995, p. 276).

NFC and TIE can be considered conceptually very similar in reflecting attraction to and engagement in challenging cognitive activity. However, there are also noteworthy differences in emphasis. NFC, as characterized above, is assessed with representative items such as “I really enjoy a task that involves coming up with new solutions to problems” or “I would prefer complex to simple problems” (Cacioppo et al. 1984, p. 307). In contrast, TIE, as “a personality trait hypothesized to relate to typical vs. maximal intellectual performance” (Goff and Ackerman 1992, p. 539), appears to represent slightly broader intellectual lifestyle preferences. Factor analyses, for instance, suggest three (Ackerman and Goff 1994), four (Dellenbach and Zimprich 2008), or five facets (Arteche et al. 2009), such as Problem-Directed Thinking, Abstract Thinking, and Reading (Ackerman and Goff 1994). Despite this, NFC and TIE have shown manifest correlations ranging from *r* = 0.78 to *r* = 0.87 (Woo et al. 2007; Powell and Nettelbeck 2014), suggesting that they are at least strongly overlapping constructs. Moreover, Mussel (2010) found a single factor to explain 67% of the variance across multiple curiosity and investment scales, and Woo et al. (2007) observed very similar associations of NFC and TIE with Big Five traits and autonomous regulation for learning. Conceptually, they can also be distinguished from similar constructs like epistemic curiosity, which appears to be more strongly characterized by a desire for knowledge and the enjoyment of learning (Litman and Spielberger 2003; Litman et al. 2005; Litman 2008). Taken together, these considerations provide a rationale for investigating NFC and TIE jointly as indicators of a common intellectual investment disposition, despite subtle conceptual differences between the two. The present study therefore focuses on both NFC and TIE, while also addressing potential differences in their relations to intelligence and executive functions. Unless addressed separately, we will refer to them jointly with “NFC/TIE” for brevity.

To integrate some of these often independently studied constructs as well as others such as intrinsic motivation (Amabile et al. 1995), Mussel (2013) proposed the Intellect framework, which includes the two dimensions process and operation. The first refers to consecutive phases in performing an action with the subcomponents seek—the desire for novel intellectual challenges—and conquer—motivational tendencies including the effort and persistence in mastering such challenges. Operation encompasses the desire to engage in intellectual activities with the subcomponents think, learn, and create, reflecting, for example, reasoning, the acquisition of new knowledge, and the ability to produce new ideas and creative outcomes (Mussel 2013, pp. 2–3). The Intellect framework thus comprises trait characteristics such as curiosity, creativeness, intellectuality, and cleverness (Mussel and Spengler 2015) with NFC and TIE being primarily located on the seek-side of process and think-side of operation.

NFC has been further conceptualized in the Elaboration-Likelihood Model of Persuasion (Petty and Cacioppo 1986). Here it is defined as a trait related to the deep elaboration of information and an important contributor to attitude change, with Cacioppo et al. (1996) summarizing empirical support for this. NFC is also associated with traits reflecting goal orientation, such as achievement striving (Fleischhauer et al. 2010, *r* = 0.44), as well as behavioral and neural indicators of cognitive effort investment (Enge et al. 2008, η^2^ = 0.12; Westbrook et al. 2013, *r* = 0.31; Mussel et al. 2016, *r* = −0.39^1^; Chevalier 2018, *r* = 0.37; Kramer et al. 2021, *r* = 0.08 and 0.13; Fleischhauer et al. 2010) and self-control under cognitive demands (Grass et al. 2019, β = 0.14). Relatedly, NFC is associated with persistence and intrinsic motivation (Lavrijsen et al. 2023, *r* = 0.37 and 0.53, respectively), while TIE is related to deep learning approaches as well as superficial leaning (Furnham et al. 2009, *r* = 0.51 and −0.19, respectively) and distractibility (Goff and Ackerman 1992, *r* = −0.35). Both NFC and TIE are also related to self-regulated learning (Woo et al. 2007, *r* = 0.56 and 0.54, respectively). In line with Mussel’s framework, NFC is further associated with enhanced explanatory thinking (Lassiter et al. 1991; β = 0.16) and information acquisition (Levin et al. 2000). It has also been found to moderate the relationship between the difficulty of a task and performance expectancies (β = −0.55), suggesting that individuals high in NFC more strongly consider task difficulty as additional information when forming their expectations (Reinhard and Dickhäuser 2009). Overall, these findings support the conceptualization of NFC/TIE as a motivational tendency to engage in effortful deliberation and search for information.

### 1.2. Intelligence

The scientific study of intelligence has been of central importance to psychology for more than a century, and debates about its nature have considerably influenced the development of the field. In early theories, a primary focus was whether intelligence reflects a single, unified capacity or a set of relatively independent abilities with Spearman (1904, 1927) being among the first to argue for a conception of intelligence as a unitary ability. Based on associations among relatively diverse cognitive tasks, he proposed intelligence to be structured into a general intelligence (*g*) factor and more specific factors (*s*) associated with specific tasks. Spearman further suggested that the g-factor reflects core processes such as the apprehension of experience, the education of relations, and the eduction of correlates, proposed to support reasoning across domains (Sternberg 2003).

This view was challenged by Thurstone (1938), who rejected the idea of a single general ability and instead suggested seven “primary mental abilities” (Thurstone 1938, p. 2), including verbal comprehension, verbal fluency, numerical ability, perceptual speed, inductive reasoning, memory, and spatial visualization. Thurstone’s multifactorial approach emphasized the distinctiveness of intellectual faculties and inspired later theories that highlighted specialized forms of intelligence (Sternberg 2003). In the following decades, further conceptualizations were proposed, ranging from hierarchical accounts attempting to reconcile Spearman and Thurstone to pluralistic models such as Gardner’s (1999) theory of Multiple Intelligences and Sternberg’s (1997) theory of Successful Intelligence. These models stressed that intelligence could not be captured by a single explanatory principle and set the stage for integrative models that acknowledged both common and specific sources of cognitive performance. An influential theoretical elaboration of such integrative approaches is the Cattell-Horn Theory of Fluid and Crystallized intelligence (Horn and Cattell 1966). However, a later integration of this model with Carroll’s (1993) Three-Stratum Model, the Cattell-Horn-Carroll (CHC) Model of Intelligence, is the model underlying most intelligence tests today (Kaufman et al. 2013).

Although many conceptions of human intelligence have been developed over the years (see Sternberg 2003), some, such as the CHC Model of Intelligence, have received wider acceptance and support than others. Concisely discussed by McGrew (2009), the CHC model is hierarchically structured: stratum III represents a general g-factor, similar to Spearman’s conception, stratum II contains a set of broad abilities, and stratum I consists of numerous narrow, task-specific abilities (Carroll 1993; McGrew 2009). Among the broad abilities at stratum II are fluid intelligence (Gf), crystallized intelligence (Gc), auditory processing (Ga), visual processing (Gv), processing speed (Gs), short-term memory (Gsm), long-term storage and retrieval (Glr), decision and reaction speed (Gt), reading and writing (Grw), and quantitative knowledge (Gq) (McGrew 2009). Of these, Gf is conceptualized as the ability to solve novel intellectual problems by relying on reasoning, an ability considered relatively independent of prior knowledge (Salthouse et al. 2008). Gc, in contrast, can be characterized as the knowledge of the world an individual has acquired and accumulated over time—for example, knowledge about politics, art, science or language.

Gf and Gc are two of the most widely discussed and examined conceptualizations of intelligence (Beauducel et al. 2001) and, as described in more detail below, influential models in the field link investment traits such as NFC/TIE explicitly to these two abilities (Cattell 1987; Ackerman 1996; Ziegler et al. 2012). By contrast, other abilities such as Gv, Ga, or Gs are not emphasized in these frameworks. Moreover, a preliminary inspection of the empirical literature on NFC/TIE and intelligence suggested that it is primarily these abilities that are examined (e.g., Ackerman 2000; Beier and Ackerman 2001; Fleischhauer et al. 2010; Hill et al. 2013; Von Stumm 2013), with, at best, only occasional investigations of the relationship with other abilities of the CHC model.

Although widely accepted, the described distinction into Gf as reasoning ability in the face of novel stimuli and Gc as acquired knowledge has also been disputed. DeYoung (2020), for instance, advocates distinguishing verbal and non-verbal intelligence to improve conceptual clarity. In the present study, however, we will rely on the traditional distinction into Gf and Gc, as characterized above. Given the strong emphasis on these two aspects in the literature on NFC/TIE and intelligence, distinguishing between verbal and non-verbal intelligence instead would have meant excluding a considerable number of composites that include tasks based on verbal material, such as letters, as measures of Gf (e.g., Ackerman et al. 2001; Salthouse et al. 2002; Ackerman and Beier 2006; Soubelet and Salthouse 2010; Von Stumm 2013). The same applies to several established measures such as the I-S-T 2000 R (Liepmann et al. 2007), in which tasks with verbal material are used to measure Gf.

### 1.3. Executive Functions

Executive functions are generally understood as “a family of top-down mental processes needed when [one has] to concentrate and pay attention, when going on automatic or relying on instinct or intuition would be ill-advised, insufficient or impossible” (Diamond 2013, p. 136). As suggested by this characterization, they play a key role not only in other cognitive abilities but also in academic, social, and psychological development (Brocki and Bohlin 2004; Diamond 2013; Moriguchi 2014).

A central debate in research on executive functions concerns the question whether they should be regarded as multiple, differentiable functions (e.g., Godefroy et al. 1999) or as a unified construct (e.g., Duncan et al. 1997). The unity and diversity paradigm (Miyake et al. 2000) integrates both of these perspectives, proposing that executive functions share a common structure while also showing unique variance reflecting specific contributions (Miyake et al. 2000; Miyake and Friedman 2012). The three components of the model and the most frequently studied executive functions are working memory updating, inhibition, and shifting (Miyake et al. 2000; Miyake and Friedman 2012; Diamond 2013; Wolff et al. 2016).

Working memory (WM) can be described as the cognitive function of keeping information in mind and mentally manipulating it. It thus enables individuals to store and flexibly add, adapt or replace information that has been present in the recent past but is no longer perceptually accessible (Diamond 2013). In a similar vein it has been described as a system for the “simultaneous processing and storage of information” (Salthouse 1990, p. 104). The updating function associated with WM is distinct from WM capacity and involves monitoring and coding incoming information for its relevance to the task at hand, as well as replacing older information when necessary (Miyake et al. 2000). WM thus encompasses more than just the passive temporary storage of information and can be differentiated from short-term memory (Oberauer et al. 2000; Cowan 2008).

Inhibition is often conceptualized as consisting of two components: resistance to distractor interference and inhibition of prepotent responses (Diamond 2013; Rey-Mermet et al. 2018). The former refers to the voluntary inhibition of attention to irrelevant stimuli, depending on current goals, and also plays a role in avoiding unwanted intrusions of prepotent mental representations into the current mental workspace (Nee and Jonides 2008; Diamond 2013). Inhibition of prepotent responses, by contrast, is defined as “the ability to deliberately inhibit dominant, automatic, or prepotent responses when necessary” (Miyake et al. 2000, p. 57).

Finally, the third executive function proposed by Miyake et al. (2000) is an individual’s ability to shift between the execution of different cognitive tasks (Monsell 2003). This function manifests, for instance, in situations in which a predeveloped plan for an action is overridden in favour of a spontaneously arising opportunity to carry out an equally or even more beneficial alternative action (Diamond 2013). Shifting thus provides the basis for a flexible adaption to changing task demands (Deák and Narasimham 2003) and is most prominently measured by paradigms in which participants must alternate between the execution of two separate tasks in response to a cue (e.g., Rogers and Monsell 1995).

This conceptualization originally proposed and first empirically tested using latent variable modelling by Miyake et al. (2000), has become an important cornerstone in research on executive functions. Subsequent work suggests, for instance, that these functions form a central part of the foundation upon which more complex, higher-order abilities such as planning, reasoning, and problem solving are built (Collins and Koechlin 2012; Diamond 2013; Schäfer et al. 2024). Over the last decades, they have received considerable attention in empirical research (Duggan and Garcia-Barrera 2015) and are studied across multiple fields, such as developmental and educational (Spiegel et al. 2021), clinical and health related research (e.g., Liu et al. 2020), as well as cognitive neuroscience (Rodríguez-Nieto et al. 2022).

### 1.4. Relating NFC/TIE to Intelligence and Executive Functions

Although NFC/TIE primarily reflects an individual’s motivation to seek out and engage in situations that involve considerable cognitive challenges—rather than the ability to succeed in such situations—several considerations nonetheless suggest that it may be associated with better performance on cognitive tests. The first line of reasoning concerns NFC/TIE-related dispositions that may manifest at the time of test taking. Within the Intellect framework, NFC and TIE are positioned in ways that suggest, on the one hand, associations with faculties such as problem solving and reasoning, as reflected in Gf, and on the other hand, associations with the acquisition of knowledge, possibly reflected in higher Gc (Mussel 2013). Associations with Gf tests scores are further suggested by the mentioned enhanced goal orientation, cognitive effort investment, self-control, persistence, intrinsic motivation, and deeper elaboration of information, all of which may influence how individuals approach standardized tests. While such considerations predict an association of NFC/TIE with actual performance, they do so only insofar as they assume a specific behaviour of an individual *during test taking.*

A different line of reasoning takes a more developmental perspective. Drawing on the Openness-Fluid-Crystallized-Intelligence (OFCI) model, based on Cattell (1987) and Ackerman (1996) and proposed by Ziegler et al. (2012) for the closely related openness to experience, NFC/TIE may over time positively influence Gf through stronger exposure to stimulating environments (environmental enrichment hypothesis). Especially during the periods of childhood and adolescence, for which research suggests strong plasticity in the formation of regions in the brain (e.g., Garlick 2002; Laube et al. 2020), such heightened cognitive stimulation may contribute to the development of higher cognitive abilities. The environmental success hypothesis, by contrast, suggests that higher Gf positively influences the development of NFC/TIE by enabling individuals to successfully manage cognitively challenging situations. This, in turn, may gradually increase interest in new intellectual challenges, resulting in higher NFC/TIE. Finally, NFC/TIE may also indirectly influence the development of Gc by increasing Gf.

Given the conceptual integration of NFC/TIE with specifically Gf and Gc in the most relevant models on the topic, as well as the strong empirical emphasis on these abilities, the present study will focus on Gf and Gc. Empirically, the relationship between these constructs has been examined for decades, with often inconsistent outcomes. Stuart-Hamilton and McDonald (2001) for instance reported only small correlations between NFC and Gc (*r* = 0.06), whereas Tidwell et al. (2000) found moderate associations with knowledge (*r* = 0.25) and verbal ability (*r* = 0.33). Stuart-Hamilton and McDonald (2001) reported Gf to be related to NFC (*r* = 0.31), Fleischhauer et al. (2010) observed a moderate correlation with Gf (*r* = 0.28) and a weaker correlation with Gc (*r* = 0.14), while Hill et al. (2013) reported that NFC predicted latent Gc (β = 0.32) as well as Gf (β = 0.40). More recently, Rudolph et al. (2018) found NFC to be related to Gf at *r* = 0.10, while Stern and Axt (2021) reported an association of *r* = 0.22 with a matrix reasoning task. Regarding aspects associated with Gc, NFC has also been found to relate to general knowledge (*r* = 0.35, Von Stumm 2013) and verbal knowledge (*r* = 0.39, Mozuraitis et al. 2016) in more recent investigations. Findings on TIE can also be considered relatively heterogeneous. Results by Zhang et al. (2018) suggest a moderate association of *r* = 0.22 with Gf, whereas, occasionally, relatively high correlations with general knowledge have been reported (*r* = 0.50, Furnham et al. 2009). Overall, associations of TIE with Gf and Gc appear to fall mostly in the range of small to moderate for Gf (*r* = 0.08–0.23) and moderate to high for Gc (*r* = 0.29–0.42; Ackerman 2000; Beier and Ackerman 2001). For both NFC and TIE, the associations reported in the literature thus vary considerably in strength, likely reflecting differences between, for instance, the examined populations and the specific measures used.

While the association of NFC/TIE with higher-order abilities such as Gf and Gc has been a primary research interest in the literature on cognitive investment traits over the last four decades, the relationship with more basic cognitive functions seems not to have been focused on. Executive functions as separable but related core abilities supporting performance on complex cognitive tasks (Miyake et al. 2000; Friedman and Miyake 2017) can be regarded as enabling higher-order abilities such as intelligence. For instance, research suggests that WM capacity and executive functioning are strongly related to Gf (Kane et al. 2005), and that there is a substantial overlap of executive functions with g (Friedman and Miyake 2017). Neuropsychological meta-analyses also suggest considerable overlap between the neural underpinnings of Gf and those of executive functions, most importantly updating (Santarnecchi et al. 2021). Moreover, executive functioning has been explicitly proposed to be a central mechanism underlying individual differences in intelligence (Engle 2018; Mashburn et al. 2024). This close relation between abilities raises the question whether associations of NFC/TIE with Gf and Gc generalize to more fundamental executive processes or are restricted to these higher-order abilities.

In the present meta-analysis, we restrict our focus to WM updating, inhibition, and shifting, as defined by Miyake et al. (2000) and further elaborated by Miyake and Friedman (2012) and Diamond (2013). This is, on the one hand, supported by the highlighted importance of this model, which also suggests an emphasis on these functions in the NFC/TIE-related empirical literature. Conceptually, on the other hand, it is specifically the three core functions that might play a role in cognitively demanding activities such as engaging in prolonged theoretical discussions, deliberating on complex issues, figuring out how something works, or systematically reading up on a new topic. These and similar NFC/TIE-related activities seem to some extent require suppressing unrelated thoughts or ideas to focus on the question at hand, manipulating temporarily remembered information while replacing it with novel input, as well as integrating information from alternating sources.

Moreover, they can be conceptually linked to a stronger expression of the traits and cognitive faculties empirically associated with NFC/TIE. For instance, enhanced goal orientation (Fleischhauer et al. 2010) should manifest in a more focused and less easily distracted approach to personal goals. And this might benefit from and, over time, increase the ability to suppress irrelevant stimuli and thoughts (Diamond 2013). Moreover, the tendency to invest cognitive effort (e.g., Westbrook et al. 2013; Mussel et al. 2016; Chevalier 2018) and persistence in working on cognitive tasks (Fleischhauer et al. 2010; Lavrijsen et al. 2023) may in these individuals over time go hand in hand with higher levels in cognitive faculties that are required for such high-load cognitive activities. Similar considerations apply to the reported associations with deep learning approaches, superficial learning (Furnham et al. 2009), distractibility (Goff and Ackerman 1992), and self-regulated learning (Woo et al. 2007). Furthermore, an association with inhibition is suggested more directly by associations of NFC with self-control (Bertrams and Dickhäuser 2012; Grass et al. 2019), to which inhibition is known to contribute (e.g., Hofmann et al. 2012; Logan et al. 1997).

Although, to our knowledge, no corresponding specific theory concerning executive functions has been developed, the above-mentioned models (Cattell 1987; Ackerman 1996; Ziegler et al. 2012) may help integrate these considerations. In that sense, high-NFC/TIE individuals should engage more often in cognitively demanding activities, thereby possibly also training cognitive functions that enable them. In contrast, a higher expression of the three core executive functions might over time lead individuals to enjoy and seek out such cognitively complex situations more often.

Together, these considerations provide a plausible explanatory pathway for how motivational dispositions such as NFC/TIE may relate to performance advantages on core executive function tasks. Moreover, executive control has been found to be more fundamental to intelligence than other, even more basic abilities such as processing speed (e.g., Mashburn et al. 2024), suggesting a focus on core executive functions alongside Gf and Gc. However, whereas the link between NFC/TIE and intelligence has been frequently examined directly, the associations with executive functions have received far less attention. Some studies addressed the relation to top-down attention (e.g., Enge et al. 2008; Enge et al. 2011; Fleischhauer et al. 2014), yet it appears that mainly Gärtner et al. (2021) systematically examined associations with updating, shifting, and inhibition. These specific findings suggest that NFC is at best only weakly associated with these three functions, the largest effect being Kendall’s τ = 0.10. Similarly, Hill et al. (2013) reported small correlations with NFC (up to *r* = 0.15), although their analyses focused on WM capacity rather than updating.

Theoretically, NFC/TIE may hence be assumed to be associated with basic executive functioning, while a very limited salient literature indicates, at best, only small associations. Concerning intelligence, conceptual and empirical considerations suggest an association with Gf and Gc. Empirically, however, the strength of effects varies considerably across studies. With these uncertainties, the present meta-analysis aims to clarify the extent of these relations.

### 1.5. The Present Study

The relationship of NFC/TIE with intelligence has been addressed in earlier meta-analyses such as Ackerman and Heggestad (1997). However, given the smaller empirical base at the time, the reported correlations relied on only one study for Gf and six for Gc. A later analysis by Von Stumm and Ackerman (2013) drew on a larger number of studies but did not investigate the associations with core executive functions. Furthermore, recent research activity suggests a growing body of literature that should now allow for more precise effect estimates. Stanek and Ones (2023) provide an exceptionally informative overview of the relationship between a wide range of personality traits and cognitive functioning. Yet their analysis appears to include publications only up to 2014 and omits several earlier high-quality primary studies specifically on NFC (e.g., Cacioppo et al. 1986; Day et al. 2007; Fleischhauer et al. 2010; Burkolter and Kluge 2012). Moreover, it primarily focuses on relationships with aspects of intelligence, memory, or processing speed, and does not consider updating, shifting, or inhibition.

To address these limitations and provide a clear and comprehensive picture of the outlined questions, we examine the available literature up to the present date and provide a meta-analytic synthesis of study results. As the literature appears to have mainly focused on the relationship of NFC/TIE with Gf on the one hand and Gc on the other, our first research question concerns the extent of associations between NFC/TIE and these two components of intelligence. We hypothesize positive associations of NFC/TIE with both Gf and Gc.

Beyond this, our study serves several additional purposes. The first is a direct comparison of NFC and TIE in their associations with cognitive functions. While research finds the constructs to overlap strongly, they also differ in their conceptualization and in measurement. This leaves a possibility that their relations to Gf, Gc, and executive functions may not be equivalent. Examining them together hence provides valuable evidence for evaluating subtle differences at the intersection of personality and cognitive ability. Furthermore, unlike previous syntheses, we address the considerable inconsistencies in effects reported in the literature by conducting moderator analyses. Identifying factors underlying these differences in effects, such as age, gender, sample characteristics, study quality, and especially specific task characteristics may provide further insights into the nomological net of NFC/TIE with respect to cognitive abilities. These moderators are discussed further below and in Supplement A. Third, we further extend prior syntheses by examining whether the observed associations with Gf and Gc also hold for core executive functions. As these functions are considered central contributors to higher-order abilities, examining whether NFC/TIE associations generalize to them or are restricted to more complex abilities will also allow us to obtain a more precise picture of how they relate to cognitive ability. Given the inconsistencies between theoretical considerations and the salient empirical findings, we did not pre-specify hypotheses regarding the association of NFC/TIE with executive functions. Taken together, these objectives may allow us to clarify the robustness, scope, and theoretical significance of NFC/TIE in relation to central cognitive functions.

## 2. Materials and Methods

### 2.1. Transparency and Openness

We followed the PRISMA 2020 guidelines for systematic reviews and meta-analyses (Page et al. 2021) as well as the APA ethical principles. Although we did not prepare a formal protocol, the study rationale, procedures, and methods were preregistered prior to the initial literature search at OSF: https://osf.io/9mvt7/overview (accessed on 26 October 2025) (a copy of the preregistration is included in the supplements). The supplements, including associated documents, research materials, data, analysis code, and results of additional analyses, can be found at https://osf.io/7n58e/overview (accessed on 26 October 2025). During the work on this project, we had to make some amendments to the preregistered procedure, such as refinements to the screening and coding tools and the estimation of multi-level models with robust variance estimation instead of two-level random-effects models. We encourage interested readers to evaluate potential implications of these changes for themselves. A detailed statement is included in Supplement K.

### 2.2. Literature Search

The main systematic search for primary studies covered a wide range of databases for published and grey literature until 28 November 2022. Databases for published literature included ERIC, ScienceDirect, PsycArticles, Psychology and Behavioral Sciences Collection, SocINDEX with Full Text, PSYNDEX, PsycINFO, International Bibliography of the Social Sciences (IBSS), Social Science Research Network (SSRN) eLibrary. Grey-literature databases included ProQuest Dissertations and Theses Global: Social Sciences, Grey Literature Database, and Search AHRQ. General search strings and details on filters and database-specific adaptions are provided in Supplement B.

References in included records were checked for other publications during screening and citations of the most cited studies on our research questions (e.g., Fleischhauer et al. 2010; Hill et al. 2013) were searched in Google Scholar from 20 June 2023 till 25 June 2023. To detect unpublished studies and incorporate upcoming work, OSF Registries (osf.io/registries) was searched. Authors in the field were first contacted on 28 July 2022. This included the email list of the DGPs (Deutsche Gesellschaft für Psychologie) as well as requests for further details on detected records and overlooked further studies in December 2023 and early 2024. Finally, earlier reviews and meta-analyses on similar research questions (see above) were screened. The most recent study included in our analysis was published in 2024 (Ruhr 2024).

### 2.3. Inclusion and Exclusion Criteria

We only included primary studies that met the following criteria. More information on criteria (b) and (c) is provided in Supplement A:

(a) The publication was written in English, German, or French.
(b) The publication examined NFC or TIE.
(c) The publication examined Gf, Gc, general intelligence^2^, or one of the above-mentioned executive functions^3^, using sufficiently described and validated performance measures.
(d) The publication was not itself a review or meta-analysis. If this criterion was not met but the publication was considered relevant to our research questions, it was later screened for relevant references.
(e) The publication sample was not drawn exclusively from clinical populations, including those characterized by psychiatric or physical disorders and diseases. We considered a publication eligible regarding this criterion if it examined data from, for example, a healthy control arm in a clinical study design.
(f) The publication quantitatively analyzed the data to obtain effect sizes such as correlation coefficients, regression coefficients, or data transferable into a Pearson correlation coefficient for the association of NFC/TIE and the respective cognitive function.

No restrictions were placed on the date of publication, and we included samples regardless of age, gender distribution, nationality, or other demographic characteristics such as occupation. We did not restrict inclusion to journal articles but considered all types of reported studies if other relevant criteria were fulfilled. This includes research reports, preprints, working papers, conference contributions, and dissertations. However, we excluded Bachelor’s and Master’s theses, as, in contrast to dissertations, these typically do not result from a lengthy, rigorously supervised research process, making biased or insufficiently controlled data more likely. Regarding (f), we excluded a record only if the necessary data were unavailable both in the main text and from additional sources (e.g., supplements, corresponding authors).

### 2.4. Screening and Coding

The identified references were first collected in an EndNote, version X9.3.3 (Hupe 2019) library to organize the database and remove duplicates. References were then listed in Excel sheets, which served as the basis for the main screening and coding procedure. Screening was divided into a title and abstract screening and a full text screening. Two screeners and coders were involved at each step: the first author and a trained, experienced research assistant working on the project.

In both the title and abstract and full-text screening, all detected references were first screened by the first screener and then by the second. Because relevant constructs were not always listed in titles, abstracts, or keywords, it was often difficult to determine relevance at this stage. For example, data on NFC/TIE or intelligence were sometimes collected as secondary measures and not mentioned in the abstract. To not fall prey to excluding potentially relevant records, we therefore did not automatically exclude a record if our constructs were not mentioned. Instead, exclusion at this stage required that the described research field, study design, or related information made it very unlikely that the study examined our constructs. Because it was difficult to determine from the title and abstract alone whether an unmentioned construct had in fact been examined, the initial interrater agreement for inclusion decisions (yes/no) in the title and abstract screening was 81%. In the individual questions, agreement for the inclusion (yes/no) of the record based on that criterion ranged from 64% in case of question (c) to 95% in question (a). Given the described liberal inclusion policy for the title and abstract screening, it is, however, unlikely that relevant studies were missed at this stage.

In the full text screening, an interrater agreement for the inclusion (yes/no) of records for coding of 99% in all questions was achieved. Unlike during screening and given the high workload during coding, coders did not extract information from each record independently. Instead, the reference list was split in half: one coder processed the first half, the other the second. References were then exchanged, and the extracted information was confirmed or corrected by the second coder. Both frequently met during the whole screening and coding process. Disagreements were resolved by examining the respective reference together and discussing its eligibility until a consensus on its inclusion or exclusion was reached.

During screening and coding, we carefully checked for studies conducted on the same datasets. In such cases, we examined whether relevant additional results or information were reported and coded only this additional information. When relevant constructs were measured multiple times, we coded the measurement judged to be least affected by confounding influences (e.g., interventions). This was usually the baseline measurement. After coding, data were transferred to separate datasheets for each meta-analysis. The exact screening questions and the codebook are included in Supplement C. References for the tests and scales used in the included studies are listed in the Supplement L and M.

### 2.5. Data Analysis

#### 2.5.1. General Models

Before specifying the below-described models, we first ensured that coefficients in primary studies reflected the same direction of association between NFC/TIE and the cognitive function, inverting them when necessary. If no Pearson correlation coefficients were reported, we derived them from available data or from other reported coefficients using formulas provided by Gilpin (1993), Walker (2003), and Peterson and Brown (2005). Fisher’s *z*-standardized correlation coefficients, sampling variances, and standard errors (SEs) were calculated and used in model estimation due to their better statistical properties (Harrer et al. 2022).

Preliminary inspection of the literature suggested substantial variation both in the populations from which samples were drawn and in the instruments. We therefore did not assume that included studies had a common population effect size, sampling error being the only source of variance (Borenstein et al. 2009). Generally, the appropriate approach would thus be a random-effects model. However, after coding it became apparent that a two-level random-effects model would not be appropriate as we found multiple publications contributing more than one sample, but also samples contributing multiple effects. In the analyses of the three executive functions, there were also cases in which a single measure contributed multiple effects because different outcomes were used. For instance, in the analysis of NFC and shifting, we used data of both the switch costs in response time and accuracy from study 2 in Fleischhauer et al. (2019). As both outcomes are generally used in shifting tasks, we included both and treated the resulting effects as nested within the task. A four-level structure with effects nested in samples nested in publications was hence assumed for the analyses of Gf, Gc, and a five-level structure in case of the three executive functions. In contrast, the data on general intelligence were simpler, allowing for a random-effects model with only sampling error and heterogeneity of true study effects as sources of variance.

Robust variance estimation (RVE) is another approach to handling dependency among effect size estimates, within which two general types of dependence structures are typically distinguished: hierarchical effects and correlated effects (Tanner-Smith et al. 2016). In case of the former, dependency arises due to effects being nested within the same unit (e.g., the same laboratory, study, publication, etc.). In the latter, dependence results from the same participants being used to estimate multiple effect sizes within the meta-analysis. Details on the model types of RVE are provided in Hedges et al. (2010), Tanner-Smith et al. (2016), and Pustejovsky and Tipton (2022). Most importantly, using a multi-level approach on dependent effects as described above comes with the drawback of assuming independent sampling errors within the modelled clusters. This assumption is, however, likely violated in our data due to multiple effects coming from the same sample. On the other hand, using a correlated-effects model based on RVE alone ignores the hierarchical structure of the data and does not provide precise estimates of variance components across levels (Tanner-Smith et al. 2016). Because we aimed to obtain precise estimates of variance at each level, neither approach alone was sufficient.

We hence adopted a strategy based on the approach proposed by Pustejovsky and Tipton (2022) and Pustejovsky (2021a, 2021b), combining a model that represents our hierarchical data with the correlated-effects RVE model (Hedges et al. 2010). RVE has the benefit of providing hypothesis tests, confidence intervals, and SEs that are highly robust to assumptions of the dependency between effects. All statistical analyses were conducted using R, version 4.4.1 (R Core Team 2024) and R Studio, version 2024.4.2.764 (Posit Team 2024). Multi-level models were estimated with the *rma.mv* function of the *metafor* package, version 4.6.0 (Viechtbauer 2010). The model on general intelligence was estimated with *rma.uni*, also from *metafor*. Based on the estimated model we then used the implementation of RVE in the *clubsandwich* package, version 0.5.11 (Pustejovsky 2024), to calculate robust SEs, hypothesis tests, and confidence intervals with the functions *coef_test* and *conf_int*. In multi-level meta-analytic models, weighting is not achieved simply by taking the inverse of the sum of variance components (τ^2^ + sampling variance in a two-level model) as in standard random-effects models. Instead, as the model implies covariation among effects, weights are calculated by considering both the covariances of effects and their sampling variances. The details of these computations are concisely presented in Viechtbauer (2024) and Pustejovsky (2020). The code for all analyses is available in the supplements.

We chose the restricted maximum likelihood (REML) estimator of heterogeneity at each level of the model, as it has been found to provide approximately unbiased heterogeneity estimates (e.g., Viechtbauer 2005; Veroniki et al. 2016). Given that it reduces the probability of false positives (e.g., Langan et al. 2019), we initially planned to apply the Knapp–Hartung adjustment (Knapp and Hartung 2003) to the calculation of SEs in our analyses. However, the *rma.mv* function used for most of the analyses does not allow for making this adjustment. Instead, it provides *p*-values based on *t*- and *F*-distributions, which yield improved inferences compared to standard methods (Viechtbauer 2010). In the *rma.mv* models, this option was applied, whereas the Knapp–Hartung adjustment was used in the *rma.uni* model on general intelligence. Moreover, the *I*^2^ statistic (Higgins and Thompson 2002) was calculated, indicating the percentage of variance attributable to variance in true effects and not sampling error (Borenstein 2019). In interpreting *I*^2^, we followed Hunter and Schmidt (1990), as cited in Assink and Wibbelink (2016), and considered heterogeneity substantial if less than 75% of the variance could be attributed to sampling error. In addition, likelihood-ratio tests were used to determine whether significant variation was present at each level of the model. The *p*-values of the two-sided tests provided by the *rma.mv* function were divided by two to obtain results for a one-sided test, as recommended by Assink and Wibbelink (2016). Results of the *Q*-test were interpreted to assess whether a significant amount of variation in true effects was present overall and prediction intervals were calculated as an index of variation in true effects across populations (Borenstein 2019). These criteria were applied to determine whether heterogeneity at each level was substantial. Finally, given the limitations in the estimation of heterogeneity, we report analyses on *all* the below described moderators, where permitted by the data (e.g., Li et al. 2015). Unless otherwise stated, the significance level in these analyses, as well as those described in the following, was α = 0.05.

Because effect size estimates are usually attenuated as a function of the measure’s reliabilities, all analyses were repeated with effect sizes corrected for attenuation. If available, reliabilities were extracted directly from the publication. If not reported in the publication, we searched for studies validating the measure. If these were also unavailable, we relied on other studies in our dataset that used the same instrument. As a last resort, we used data from very similar instruments (e.g., a different but comparable version of Raven’s Matrices). We hence did not rely on a broad average of the reliability (e.g., *r*_XX_ = 0.78 for Gf measures, Stanek and Ones 2023) for all tests. Instead, our correction was based either on the actual reliabilities of a specific test in the sample or the most precise estimate of it. Details for each instrument are provided in Supplement H (Tables S20–S25). The correlation coefficients were corrected for attenuation by dividing them by the square root of the product of the measures’ reliabilities. The corrected coefficients were then Fisher’s *z*-standardized, and sampling error variances were calculated using the second-order delta method (Zhang and Wang 2024).

#### 2.5.2. Outliers and Influential Cases

Estimates in meta-analyses can be biased by extreme effect sizes (Harrer et al. 2022). To avoid excluding cases that may provide important insights, we only considered excluding studies when they were both outliers and influential on the model. Two indicators were used to identify outliers. The first was a studentized deleted residual greater than 1.96 (Viechtbauer and Cheung 2010) and the second a confidence interval of the primary study’s effect size that did not overlap with the confidence interval of the pooled effect (Harrer et al. 2022). To detect highly influential studies, we initially planned to consider several indicators, such as the covariance ratio, Cook’s distance, DFFITS, hat values, and study weights (Harrer et al. 2022). However, we had to drop several diagnostics of influential cases as the *metafor* package only provides functions for Cook’s distance, hat values, and DFBETAs in complex meta-analytic models. Cook’s distance larger than χ^2^(p’, 0.5) (with p’ = degrees of freedom) (Viechtbauer and Cheung 2010), hat values larger than 3 × 1/*e* (with *e* = number of effects) (Harrer et al. 2022), and DFBETAs (Viechtbauer and Cheung 2010) larger than 1 were considered potentially influential. Sensitivity analyses were conducted for both outliers and influential cases.

#### 2.5.3. Moderator and Meta-Regression Analyses

We planned to conduct moderator and meta-regression analyses of variables that might help explain potential heterogeneity in the findings, provided that the number of effects was large enough. As a rule of thumb, at least 10 studies should be included in an analysis (Schwarzer et al. 2015). However, to avoid reduced power and to maintain interpretability, we additionally required at least four studies per group and conducted analyses only if this criterion was met.

As it was uncertain which variables could be obtained from enough of the primary studies, and because the literature did not clearly suggest specific variables of particular importance, we did not prespecify the exact moderator and regression analyses. The analyses conducted here can therefore be considered exploratory. More detailed information on each moderator is provided in Supplement A. The moderators we examined include the percentage of female participants, the mean age, the risk of bias in a sample, coded using the 8-item Risk of Bias Utilized for Surveys Tool (ROBUST) (Nudelman and Otto 2020) with values ranging from 0 to 8, samples consisting exclusively of college students vs. other participants, simultaneous vs. non-simultaneous measurement of constructs (the same day vs. more than one day apart), controlled (laboratory) vs. uncontrolled (e.g., at home) measurement environment, characteristics of the tasks that were used to measure a cognitive function (e.g., tasks measuring inductive, deductive, spatial, or mixed reasoning abilities), publication year or, in case of dissertations, the year in which a work was finished^4^, as well as publication status (unpublished vs. published vs. dissertation). Finally, given the high similarity of NFC and TIE, we considered it appropriate to conduct meta-analyses including measures of both traits. However, because differences in their associations with the examined outcomes cannot be ruled out, moderator analyses were conducted to estimate separate effects for NFC and TIE.

Each moderator was examined at the level in the analysis at which it divided effects in subgroups (e.g., sample, publication). Moderator effects and group differences were tested for significance using robust Wald-tests implemented in *clubsandwich* and *metafor*. When multiple comparisons were conducted, *p*-values were adjusted using the Holm correction. The significance of residual heterogeneity was determined with a *Q*-test.

#### 2.5.4. Assessment of Publication Bias

We used funnel plots with the *z*-standardized SE of the effect to visually examine asymmetry, potentially indicating missing studies, and tested for statistical significance with Egger’s regression test (Egger et al. 1997). This was implemented by regressing the observed effect sizes onto the inverse of the SE (precision). A significant intercept in the model was interpreted as evidence of asymmetry (Harrer et al. 2022). In addition, we conducted PET-PEESE analyses for SEs (Stanley and Doucouliagos 2014) to adjust for potential small-study effects by including the SE and SE^2^, respectively, as predictors. A model using the SE (PET) generally performs better when the true effect is zero, whereas a model using SE^2^ (PEESE) yields better results when it is nonzero. Accordingly, the intercept of the PET model was interpreted when the true effect was assumed to be zero, and the intercept of the PEESE model in case of a significant (one-sided) test of the effect being larger than zero (Harrer et al. 2022). As the PET-PEESE analysis can lead to invalid results when based on a small number of effects, we report results but interpret them carefully if fewer than 20 effects were available and heterogeneity in true effects (*I*^2^) exceeded 80% (Stanley 2017).

We calculated *p*-values for each effect identified in the literature and then conducted a *p*-curve analysis (Simonsohn et al. 2014) by first plotting *p*-values < .05. On visual inspection, data in which *p*-hacking is present typically shows more cases just below the .05 threshold than expected, as this is the conventional cutoff for statistical significance. We further examined the distribution of *p*-values by splitting the *p*-values < .05 (full *p*-curve) into those smaller and those larger than .025 and applied a binomial test to examine if the distribution of values in both groups is consistent with the hypothesis that high and small *p*-values are equally likely (Harrer et al. 2022). Additionally, we split the *p*-values < .025 (half *p*-curve) into those smaller and those larger than .0125 to test for “ambitious *p*-hacking” (Simonsohn et al. 2015, p. 1149). As this method comes with the drawback of dichotomizing *p*-value data, we further analyzed the distribution by following a procedure described by Harrer et al. (2022, p. 258). For each effect, a “*pp*-value” was calculated by multiplying the *p*-value by 20, log-transforming the result, summing across studies, and multiplying by −2. This test statistic follows a χ^2^ statistic with 2 × *e* (*e* = number of effects) degrees of freedom and was used to examine how likely the data was under the hypothesis of right skewness and hence no effect.

In addition, we applied the trim-and-fill technique (Duval 2005; Duval and Tweedie 2000), which examines asymmetry using a funnel plot and provides adjustments for this potential bias (Shi and Lin 2019). However, as it seems that no implementation of this technique currently exists for models with more than two levels, we had to drop the analysis on these models. The same applies to the fail-safe method (Carson et al. 1990). These techniques were hence only used on the model on general intelligence estimated with *rma.uni*.

## 3. Results

### 3.1. Intelligence, Executive Functions, and NFC/TIE

Figure 1 displays the PRISMA diagram of the identified, included, and excluded records at each step of the procedure. Table 1 contains the results of the main analyses. References of the included studies are provided in Supplement O, main descriptive statistics are provided in Supplement E (Tables S1–S6), and Figure 2 displays the distribution of effects by publication year. Forest plots for the analyses are included in Supplement D (Figures S1–S6). Results of the same analyses corrected for attenuation are included in Supplement J (Figures S26–S32).

The analysis on NFC/TIE and Gf included 76 effects, nested within 69 samples, and 61 publications with *N* = 25,367 participants. It yielded a highly significant overall effect of *r* = 0.18 (*p* < .001, 95% CI [0.15, 0.20]). However, we also observed a highly significant amount of heterogeneity between effects (*Q*(75) = 232.81, *p* < .001) and a relatively wide prediction interval (95% PI [−0.01, 0.35]). This appears to be mainly a result of differences between samples, as 72.89% of the variance (τ = 0.094) was attributable to this. In the analysis on NFC/TIE and Gc, 65 effects were nested in 56 samples, coming from 51 publications with *N* = 14,651. Here we also observed a highly significant association of NFC/TIE with Gc (*r* = 0.26, *p* < .001, 95% CI [0.23, 0.29]), but also a significant amount of heterogeneity in effects (*Q*(64) = 179.71, *p* < .001) and a large prediction interval (95% PI [0.08, 0.43]). Variation appears to be mainly due to differences between publication clusters and between tasks nested in samples (τ = 0.086; *I*^2^ = 58.19% and τ = 0.030; *I*^2^ = 6.70%, respectively). NFC/TIE and general intelligence were also significantly associated (*r* = 0.23, *p* < .001, 95% CI [0.18, 0.28]) across *N* = 8479 participants. Heterogeneity was again highly significant (*Q*(23) = 78.99, *p* < .001; τ = 0.108; *I*^2^ = 78.10%), and the prediction interval was broad (95% PI [0.01, 0.44]). In these analyses, the only significant level-specific heterogeneity was between-publication differences in the association of NFC/TIE with general intelligence (see Table 1).

In the analyses on NFC/TIE and executive functions, we found NFC/TIE to be highly significantly related to WM (*r* = 0.14, *p* < .001, 95% CI [0.10, 0.18]). This analysis included both measures primarily targeting WM capacity and those targeting the updating function (see moderator analyses). In total, 50 effects from 45 different tasks were nested within 41 samples and 36 publications, with *N* = 7005 participants. As in the other analyses, there was significant heterogeneity between effects (*Q*(49) = 93.16, *p* < .001), also reflected in the prediction interval (95% PI [−0.06, 0.33]). Most of the variance was attributable to differences between samples (τ = 0.078; *I*^2^ = 36.13%). Note that no effects of TIE with inhibition or shifting were available. Results are hence based solely on NFC. For inhibition, the total effect was *r* = 0.04 (*p* = .077, 95% CI [−0.01, 0.09]) with 12 different publications, contributing 13 different samples, 19 different tasks, 21 effects, and *N* = 2895 participants. We did not observe significant heterogeneity between effects (*Q*(20) = 27.92, *p* = .111) with 16.91% (τ = 0.040) of the variance being attributable to differences between tasks within samples (95% PI [−0.06, 0.14]).

The analysis of NFC and shifting contained *N* = 1727 participants from 8 publications, containing 9 different samples, 10 different tasks, and 13 effects. The overall effect was not significant (*r* = 0.01, *p* =.642, 95% CI [−0.05, 0.07]) and no significant heterogeneity was observed (*Q*(12) = 9.95, *p* = .620, 95% PI [−0.06, 0.08]). Only 4.42% (τ = 0.019) of the variance was attributable to between-sample differences, with the remainder due to sampling error. In none of the analyses on executive functions did we observe a significant amount of heterogeneity attributable to differences at any level in the analysis.

In an overall analysis including effects across all examined cognitive functions, the association of NFC/TIE with Gc was significantly stronger than with Gf (*F*(1, 37.01) = 18.22, *p* < .001), WM (*F*(1, 45.78) = 27.70, *p* < .001), inhibition (*F*(1, 11.87) = 66.22, *p* < .001), and shifting (*F*(1, 9.15) = 96.74, *p* < .001), but not general intelligence (*F*(1, 24.20) = 0.45, *p* = .507). Gf was less strongly related to NFC/TIE than general intelligence (*F*(1, 29.74) = 6.75, *p* = .014), but more strongly than inhibition (*F*(1, 11.36) = 26.06, *p* < .001) and shifting (*F*(1, 8.82) = 45.71, *p* < .001). There were no significant differences in effect between WM and Gf (*F*(1, 46.15) = 2.58, *p* = .115). The association with general intelligence was significantly stronger than that with inhibition (*F*(1, 16.40) = 38.82, *p* < .001), shifting (*F*(1, 12.39) = 55.05, *p* < .001), and WM (*F*(1, 44.46) = 12.29, *p* = .001). Finally, significant differences were found between the associations with WM and inhibition (*F*(1, 11.41) = 14.81, *p* = .003) and WM and shifting (*F*(1, 8.31) = 30.45, *p* < .001), but not between shifting and inhibition (*F*(1, 8.06) = 0.84, *p* = .386).

### 3.2. Moderator Analyses

Table 2, Table 3, Table 4, Table 5, Table 6 and Table 7 summarize the results for the moderator analyses. Descriptives on the moderator variables can be found in Supplement F (Tables S7–S13). For some moderators, such as the percentage of female participants and the mean age, values were missing. In these cases, we first estimated the model as specified before but with missing cases excluded and then conducted the moderator analysis to allow comparisons of heterogeneity estimates before and after including the variable.

In the model examining Gf and NFC/TIE (Table 2), the moderator analysis comparing effects from NFC and TIE scales revealed a significant moderating effect (*F*(1, 12.10) = 5.04, *p* = .044). The association was somewhat stronger for NFC with Gf (*r* = 0.19, *p* < .001, 95% CI [0.16, 0.22]) than for TIE (*r* = 0.12, *p* = .003, 95% CI [0.05, 0.18]). No further significant effects were detected, but some interesting patterns emerged regarding the characteristics of the tasks and scales used to measure Gf and NFC/TIE. We found comparable and significant associations of NFC/TIE with measures mainly targeting inductive reasoning, spatial reasoning, and mixed reasoning abilities (ranging from *r* = 0.16 to 0.19). In contrast, associations with tasks measuring mainly deductive reasoning were still significant but lower (*r* = 0.10, *p* = .024, 95% CI [0.02, 0.19]). Regarding the content of the reasoning operation (Amthauer et al. 2001), measures using predominantly figural material (e.g., Raven’s Progressive Matrices; Raven 2000) or mental-rotation tasks (e.g., MRT-A; Vandenberg and Kuse 1978) and measures using mixed material showed similar associations (ranging from *r* = 0.18 to 0.19). The association with measures using mainly verbal material (e.g., Employee Aptitude Survey; Ruch and Ruch 1980) was lower in comparison (*r* = 0.11, *p* = .005, 95% CI [0.05, 0.16]), although not statistically significant. Finally, there was a trend towards significance for risk of bias in the sample as a predictor of the effect (*b*_1_ = 0.027, β_1_ = 0.03, *p* = .070, 95% CI [0.00, 0.06]).

In the model on NFC/TIE and Gc (Table 3), publication year emerged as a significant predictor of the effect (*b*_1_ = −0.006, β_1_ = −0.04, *p* = .010, 95% CI [−0.08, −0.001]). Furthermore, effects from NFC scales (*r* = 0.24, *p* < .001, 95% CI [0.20, 0.27]) were significantly lower (*F*(1, 13.10) = 10.70, *p* = .006) than those from TIE scales (*r* = 0.35, *p* < .001, 95% CI [0.28, 0.42]). There were stronger associations of NFC/TIE with general knowledge (*r* = 0.29, *p* < .001, 95% CI [0.23, 0.35]), followed by mixed aspects of Gc (*r* = 0.26, *p* < .001, 95% CI [0.20, 0.34]), verbal knowledge (*r* = 0.25, *p* < .001, 95% CI [0.21, 0.29]), and both verbal knowledge and reasoning (*r* = 0.24, *p* = .001, 95% CI [0.14, 0.34]). However, these differences did not become significant. As publication year and the scale used to measure NFC/TIE turned out to be significant moderators, we followed a suggestion by Hox (2010), as cited in Assink and Wibbelink (2016), and specified a further meta-regression model including both variables. Overall heterogeneity in the model remained significant (*Q*(62) = 135.39, *p* < .001), as did the effects of the NFC/TIE scale moderator (*F*(1, 14.50) = 6.30, *p* = .025) and publication year (*b*_1_ = −0.004, β_1_ = −0.03, *p* = .043, 95% CI [−0.06, 0.00]).

**Figure 3:**
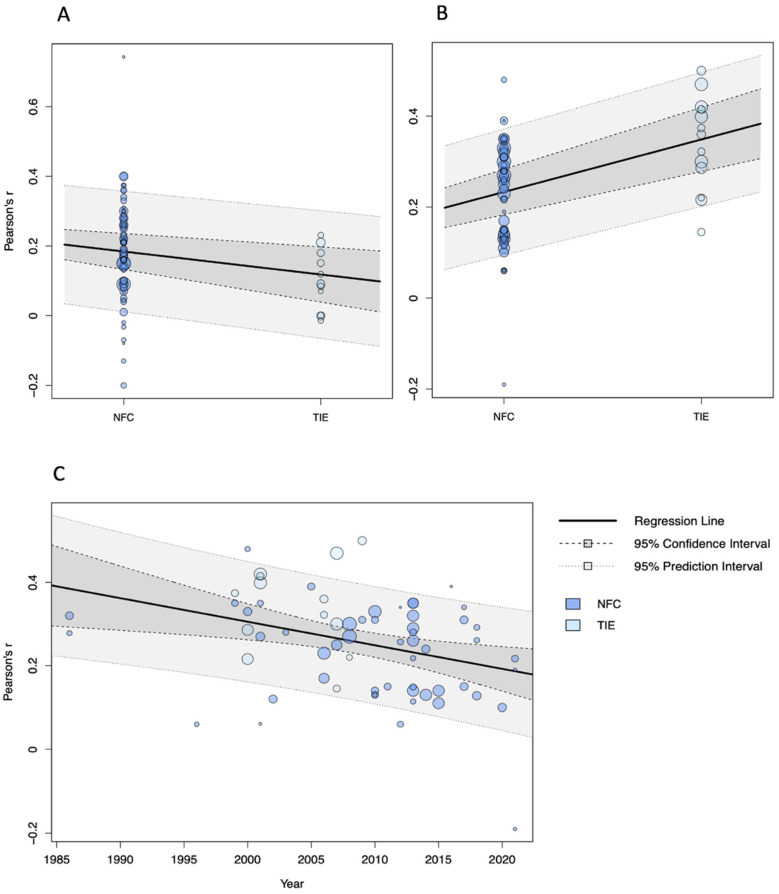
Meta-Analytic Bubble Plots for Moderator Analyses on the Association of NFC/TIE with Gf and Gc. NFC = need for cognition; TIE = typical intellectual engagement; bubble size represents the weight assigned to each effect; (**A**): moderating effect of NFC/TIE scale on association of NFC/TIE with Gf; (**B**): moderating effect of NFC/TIE scale on association of NFC/TIE with Gc; (**C**): moderating effect of publication year on association of NFC/TIE with Gc.

Interestingly, after controlling for publication year, the associations of NFC (*r* = 0.33, *p* < .001, 95% CI [0.23, 0.43]) and TIE (*r* = 0.42, *p* < .001, 95% CI [0.33, 0.52]) with Gc increased considerably. No further significant effects or statistical tendencies were observed. Figure 3 presents meta-analytic bubble plots of the significant moderator effects found in the analyses of NFC/TIE with Gf and Gc, respectively. In the model on NFC/TIE and general intelligence (Table 4), no significant moderator effects or other notable findings emerged.

In the analysis on NFC/TIE and WM (Table 5), no significant moderator effects were found for any of the examined variables. However, a notable tendency was that NFC/TIE was considerably more strongly related to WM capacity tasks (*r* = 0.15, *p* < .001, 95% CI [0.10, 0.20]) than to updating tasks (*r* = 0.08, *p* = .111, 95% CI [−0.03, 0.18]). In addition, more recent publications reported smaller effects (*b*_1_ = −0.007, β_1_ = −0.05, *p* = .099, 95% CI [−0.10, 0.01]). Residual heterogeneity in all four models on Gf, Gc, general intelligence, and WM remained significant after the inclusion of each moderator. Similarly, no significant moderator effects were found in the model on NFC and inhibition (Table 6). The only notable tendency was a somewhat larger effect for tasks primarily measuring interference control (*r* = 0.07, *p* = .054, 95% CI [0.00, 0.15]) than for those measuring response inhibition (*r* = 0.01, *p* = .465, 95% CI [−0.04, 0.06]). For this moderator analysis, one effect (Ksiazkiewicz 2015) was removed from the original model, as the measure comprised multiple inhibition tasks, targeting both response inhibition and interference control. Interestingly, while the total effect for NFC and inhibition was not significant in the original model, it did reach significance in this reduced model (*r* = 0.05, *p* = .041, 95% CI [0.00, 0.10]).

In the model on NFC and shifting (Table 7), no heterogeneity of effects was observed. Non-significant tendencies were, for example, a positive association with mean age (*b*_1_ = 0.005, β_1_ = 0.05, *p* = 0.068, 95% CI [−0.01, 0.10]) and a somewhat larger effect in non-college samples (*r* = 0.06, *p* = .138, 95% CI [−0.04, 0.16]) compared to samples consisting exclusively of college students (*r* = −0.01, *p* = .668, 95% CI [−0.12, 0.09]). Outlier and influence diagnostics across all analyses revealed several potentially outlying or influential effects, yet none that were both. Sensitivity analyses for these cases in both main- and moderator analyses indicated only small changes in effect sizes, most of which were <0.02. Except for the above-mentioned case of inhibition, none of these changes altered the significance of the results.

### 3.3. Publication Bias

Visual inspection of the funnel plots on the model examining Gf revealed potential asymmetry, partly driven by studies such as the first experiment in Parry and Stuart-Hamilton (2010, *r* = 0.74). Funnel plots for Gc and general intelligence also showed asymmetry, though less pronounced than in case of Gf. The plot for inhibition displayed a particularly strong pattern, with especially two studies (Sandra and Otto 2018, *r* = 0.37; Svedholm and Lindeman 2013, Study 1, *r* = 0.35) standing out. Finally, in the plot on shifting, asymmetry was mainly caused by one effect (*r* = −0.21) from the first experiment in (Vermeylen et al. 2019). By contrast, the plot on WM appeared relatively symmetrical. The trim-and-fill method applied to the model on NFC/TIE and general intelligence produced results similar to the main analysis. Egger’s regression test indicated significant asymmetry in the models on general intelligence (β_0_ = 0.26, *p* < .001, 95% CI [0.15, 0.36]), Gf (β_0_ = 0.17, *p* < .001, 95% CI [0.11, 0.22]), Gc (β_0_ = 0.24, *p* < .001, 95% CI [0.17, 0.31]), but not in those on WM (β_0_ = 0.03, *p* = .596, 95% CI [−0.10, 0.16]), inhibition (β_0_ = 0.07, *p* = .524, 95% CI [−0.21, 0.35]), and shifting (β_0_ = −0.12, *p* = .086, 95% CI [−0.27, 0.03]). All plots can be found in Supplement I (Figures S1–S13).

PET-PEESE analyses yielded corrected effects of *r* = 0.16 (*p* < .001, 95% CI [0.11, 0.21]) in the model on Gf, *r* = 0.28 (*p* < .001, 95% CI [0.22, 0.34]) for Gc, *r* = 0.21 (*p* < .001, 95% CI [0.11, 0.31]) for general intelligence, and *r* = 0.18 (*p* < .001, 95% CI [0.12, 0.24]) in the general model on WM. The corrected effect was *r* = 0.19 (*p* < .001, 95% CI [0.13, 0.25]) for WM capacity, *r* = 0.12 (*p* = .034, 95% CI [0.01, 0.24]) for WM updating, *r* = −0.09 (*p* = .557, 95% CI [−0.45, 0.28]) for inhibition, and *r* = 0.09 (*p* = .093, 95% CI [−0.04, 0.23]) for shifting.

The distribution of *p*-values for all models except shifting (which had no *p*-values < .05) is provided in Supplement I (Figures S14–S18). There was no indication of *p*-hacking in the models on Gf, Gc, general intelligence, and WM, as both the binomial tests on the full and the half *p*-curves were highly significant (*p* < .001). By contrast, the binomial test was not significant in case of the full (*p* = .688) and half *p*-curve (*p* = .250) in the model on inhibition. The χ^2^ test was significant in the analysis on Gf on both the full (χ^2^(100, *N* = 50) = 1100.95, *p* < .001) and the half (χ^2^(84, *N* = 42) = 1093.89, *p* < .001) *p*-curve data. The same was true for Gc (full *p*-curve: χ^2^(96, *N* = 48) = 1236.78, *p* < .001; half *p*-curve: χ^2^(88, *N* = 44) = 1234.54, *p* < .001), general intelligence (full *p*-curve: χ^2^(38, *N* = 19) = 455.34, *p* < .001; half *p*-curve: χ^2^(36, *N* = 18) = 454.06, *p* < .001), and WM (full *p*-curve: χ^2^(34, *N* = 17) = 162.99, *p* < .001; half *p*-curve: χ^2^(32, *N* = 16) = 161.68, *p* < .001). In the model on inhibition, the χ^2^ test was not significant (full *p*-curve: χ^2^(8, *N* = 4) = 8.58, *p* = .379; half *p*-curve: χ^2^(4, *N* = 2) = 6.62, *p* = .157). Results of the fail-safe method in the model on general intelligence indicate that 326 null effects would be required to render the result non-significant at a significance level of 0.05, suggesting that the findings are highly robust to potentially missing data.

In addition to these meta-analytic results, we obtained correlational data from single tasks that were also used to form a composite score reflecting, for example, Gf. In these cases, we only included the correlations of NFC/TIE with the composite in the meta-analysis. For interested readers, the effects from individual tasks are included in Supplement J.

## 4. Discussion

### 4.1. Main Findings

In the present study, we conducted six distinct meta-analyses to examine the relationship between NFC/TIE and intelligence as well as executive functions. In doing so, we pursued several goals. A first was to analyze for the first time the considerable inconsistencies in associations of NFC and TIE with Gf and Gc using moderator analyses. Second, we investigated their relation to core executive functions. As these are considered to underlie higher-order abilities, this provides insights into whether the relationship with intelligence also generalizes to more basic cognitive functions. Finally, we analyzed potential differences in how NFC and TIE as two highly similar constructs at the intersection of personality and cognitive ability relate to cognitive functions.

Overall, our analysis included 76 effects (*N* = 25,367) for Gf, 65 effects (*N* = 14,651) for Gc, and 24 effects for general intelligence (*N* = 8479). Compared to previous meta-analyses on Gf and Gc (Ackerman and Heggestad 1997; Von Stumm and Ackerman 2013; Stanek and Ones 2023), our results are thus based on a substantially larger number of studies and participants, allowing for more robust conclusions. The other three analyses on the association with executive functions, in contrast, were based on a smaller empirical base. This was particularly true for shifting (*N* = 1727, *e* = 13) and inhibition (*N* = 2895, *e* = 21). The overall analysis on WM included a total sample size of *N* = 7005 (*e* = 50), with *N* = 5921 (*e* = 40) pertaining to WM capacity and *N* = 1084 (*e* = 10) to updating. Although fewer studies examined the relationship of NFC/TIE with these functions, our study provides the first meta-analytic results in this domain.

Regarding Gf, primary studies have reported widely varying results, ranging from null effects (e.g., Bagby et al. 1986) to substantial associations (e.g., Powell and Nettelbeck 2014). Our findings suggest that NFC/TIE and Gf are indeed associated, though only to a small-to-moderate extent (*r* = 0.18). The estimate of the PET-PEESE correction for small study effects (*r* = 0.16) suggest, at most, a slight influence of small-study effects. In the analysis on general intelligence and NFC/TIE, the initially found effect was somewhat higher (*r* = 0.23), with the corrected estimate from the PET-PEESE analysis (*r* = 0.21) again suggesting only small biasing influences. Finally, the association with Gc was found to be stronger than with Gf (*r* = 0.26) and even higher when correcting for small-study effects (*r* = 0.28). As suggested by the *F*-test conducted on this difference (*F*(1, 37.01) = 18.22, *p* < .001), NFC/TIE is significantly more strongly associated with aspects of intelligence that reflect accumulated knowledge than with the ability to solve novel intellectual problems.

As mentioned above, in Mussel’s Intellect framework NFC and TIE are located on the seek-side of the process dimension and primarily on the think-side of the operation dimension, as opposed to the learn-side corresponding more to Gc (Mussel 2013). This suggests that NFC/TIE should be more strongly associated with Gf than with Gc. Furthermore, high-NFC individuals have been characterized as process-oriented rather than outcome-oriented (e.g., Cacioppo et al. 1996; Day et al. 2007), which might suggest that the accumulated knowledge resulting from their intellectual activities is of relatively little importance. While both of our hypotheses regarding positive associations were confirmed, our findings do not support this.

Given that our analyses are based on cross-sectional data, we cannot make strong claims about developmental explanations of these associations or fully evaluate theoretical accounts of the interplay proposed by Cattell (1987), Ackerman (1996), and, in a unified model, Ziegler et al. (2012). Nevertheless, an individual’s current level of Gc has been suggested to be a function of the individual’s prior levels of Gf as well as its investments into learning and intellectual pursuits (Ackerman 1996). High levels of Gf enable individuals to engage in situations in which there is potential to learn, and when combined with a stronger tendency to invest time in such activities, this may result in greater accumulated knowledge and thus higher Gc (Cattell 1987). Although intellectual activities during development might also contribute to increases in Gf, our findings may be explained by the idea that genuine cognitive improvements due to intellectual activities require more substantial long-term changes in brain functioning. By contrast, knowledge accumulated in the process may be more readily retained. Individuals high in NFC/TIE may therefore indeed incline more to the think-side of operation than the learn-side, reflecting constructs like goal orientation and interest-type curiosity (Mussel 2013). Yet the process of thinking deeply still appears to contribute more strongly to greater knowledge acquisition than to substantial improvements in Gf.

A general observation from our results is that the effects are, at best, of moderate magnitude. One explanation for this might be that traits such as NFC and TIE are commonly conceptualized as reflecting *typical* behaviour, while intelligence tests are designed to measure *peak* levels of cognitive performance (e.g., DeYoung 2020). This might suggest that personality and intelligence are two relatively distinct domains, possibly providing an explanation for only small-to-moderate associations. As critically pointed out by DeYoung (2020), however, this apparent distinction in abilities and personality traits is questionable given the possibility of measuring personality traits with ability tests, the strongly suggested influence of abilities on typical behaviour, and the importance of accounting for measurement variance. Regardless of whether these domains are truly distinct, our findings may partly reflect the fact that the peak performance targeted by intelligence tests does not necessarily represent an individual’s typical cognitive behaviour. Using measures of typical cognitive performance instead would allow targeting the domain of everyday behaviour of an individual with both the personality scale and the cognitive test. Although the literature mostly reports results from tests of maximal performance, measures of typical cognitive performance might yield higher associations with NFC/TIE scales.

Further explanations specifically concern characteristics of high-NFC/TIE individuals which may affect performance on a test without necessarily reflecting a higher expression on the construct level. As suggested above, higher cognitive effort investment and motivation to engage in novel intellectual challenges (e.g., Fleischhauer et al. 2010; Westbrook et al. 2013; Chevalier 2018) might be expected to improve performance on Gf tests relative to low-NFC/TIE individuals. However, Gf tests often pose a combination of power and speed demands (e.g., the I-S-T 2000 R; Amthauer et al. 2001) and thus require not only responding correctly but also as quickly as possible. The assumed typical behaviour of high-NFC/TIE individuals, such as the thorough engagement with intellectual problems, the evaluation of arguments, does usually not occur under strict time constraints. What characterizes these individuals, and what their behaviour to some extent seems to require, is perseverance and accuracy in evaluating information and drawing conclusions, irrespective of the time needed. The time constraints often imposed in Gf tests might therefore prevent the deeper engagement characteristic of high-NFC/TIE individuals from coming into full effect. By contrast, higher effort investment and motivation are arguably even less relevant in measures of Gc. In such tests, one either knows the answer or does not, leaving little room for performance gains through additional effort. Instead, results of Gc tests may be a better reflection of the *typical* behaviour of these individuals, as the time and effort they have invested in accumulating knowledge over years is likely reflected in their performance. Overall, this might have contributed to higher associations with Gc than with Gf.

The present findings differ somewhat from results reported in previous meta-analyses. The associations of general intelligence measures with scales measuring traits similar to NFC/TIE reported in Stanek and Ones (2023) (*r* = 0.26 with intellect to 0.28 with NFC) are slightly higher than the *r* = 0.23 found here. Those with verbal ability (*r* = 0.21 and 0.28 with intellect and NFC, respectively) and Gf (*r* = 0.17. and 0.21 with intellect and NFC, respectively), on the other hand, are overall comparable to *r* = 0.26 with Gc and *r* = 0.18 with Gf reported here. This suggests subtle differences, possibly reflecting variations in the measures (e.g., composites, inventories, or single tasks) and differences in the included studies. Note, for instance, that—possibly due to the stronger focus on identifying studies examining NFC/TIE—several studies were included here that apparently were not included in Stanek and Ones (e.g., Day et al. 2007; Fleischhauer et al. 2010).

The main findings of our study also provide novel insights, as we meta-analytically examined for the first time the associations of executive functions with NFC/TIE. We will address the conducted moderator analyses below, but as evident in the descriptions (Supplement F, Tables S7–S13), the main analysis on WM was dominated by tasks that primarily measure WM capacity. Similarly to explanations expressed above regarding Gf, the relationship between WM capacity and NFC/TIE may derive from the demands that cognitively challenging endeavours place on individuals high in NFC/TIE. As these individuals are highly motivated to engage in such activities, they may do so more frequently than others and, over time, become more skilled at storing information needed for a given task. Echoing the environmental success hypothesis (Ziegler et al. 2012), individuals who develop these skills may, in turn, become more interested in cognitively demanding activity.

However, common conceptualizations of core executive functions comprise primarily the updating function of WM, together with shifting and inhibition. Although one might assume that these also play a role in complex challenges, our results do not suggest a generalization of associations with intelligence to core executive functions. Although we found a very small association of NFC with WM updating (*r* = 0.08), inhibition was related to NFC at *r* = 0.04 and the relation with shifting was essentially a null effect (*r* = 0.01). None of these effects became significant. However, the picture changes somewhat when considering the corrected estimates from the PET-PEESE analysis. Although neither effect turned out significant, the corrected association was *r* = 0.09 (*p* = .093) for NFC and shifting, *r* = 0.12 (*p* = .034) for updating, and *r* = −0.09 (*p* = .557) for inhibition. Given the comparatively small number of publications, especially for shifting (*k* = 8), these effects are more difficult to interpret. The differences from the original effects suggest that both findings may have been influenced by small samples and thus large SEs. Similarly to the expressed ideas on WM, an assumption that conflicts with these small associations is that such abilities are required for sustained engagement in complex intellectual problems, an activity high-NFC individuals might have been thought to lean to. If these abilities are indeed relevant, however, the motivation to engage in such activities and the ability to be successful may come apart. As the original result and the corrected estimate of the PET-PEESE analysis diverge considerably, it remains unclear which provides the more accurate reflection. Given the limitations of the PET-PEESE analysis regarding heterogeneity and sample size, a cautious interpretation is that NFC is at best only weakly associated with core executive functions.

As with Gf tasks, higher associations might have been expected simply because high-NFC/TIE individuals tend to invest more cognitive effort, display stronger goal orientation, and show greater motivation toward cognitive tasks, irrespective of construct-level relationships with executive functions. The weak associations found here might, however, be explained by the fact that tasks such as the number–letter task (Rogers and Monsell 1995) or flanker task (Friedman and Miyake 2004) arguably do not represent interesting cognitive challenges for high-NFC/TIE individuals. In contrast, reasoning tasks or philosophical problems require intense deliberation and may represent more complex and engaging puzzles.

Regarding the presence of small study effects and the possibility of publication bias, our results indicate funnel plot asymmetry in some models, suggesting that a few comparatively small studies contributed unexpectedly large effects. However, this alone does not support the assumption of systematic publication bias. Moreover, the distribution of *p*-values is arguably a more realistic indicator of bias given the importance of significance thresholds. With the exception of the model on inhibition, our results indicated no such bias in the data. Yet, as this distribution was based on only four *p*-values, the reliability of these findings may be questioned.

### 4.2. Moderator Analyses

Our findings on NFC/TIE confirm the impression of a substantial heterogeneity in the associations to cognitive functions, as suggested by significant *Q*-tests in the models on Gf, Gc, general intelligence, and WM. In only some cases, however, did the extent of heterogeneity attributable to *individual* levels in the data meet the above-specified criteria. Given the limitations associated with some of the diagnostic tools discussed below, we nonetheless conducted moderator analyses on all models to examine potential influences on the relationships. Here, we focus on the most relevant findings.

First, we tested whether scales measuring NFC yielded different effects than those measuring TIE. To do so, explicitly labelled NFC scales and those based on the same items (e.g., Mental Efforts Tolerance Questionnaire; Dornic et al. 1991) were grouped together and compared to TIE scales. While results were relatively heterogenous, the moderator effects in case of Gf and Gc are noteworthy, as both turned out significant. Interestingly, while NFC was more strongly associated with Gf (*r* = 0.19) than TIE (*r* = 0.12), TIE was more strongly related to Gc (*r* = 0.35) than NFC (*r* = 0.24). Because publication year was also a significant predictor in the analysis on Gc, we estimated a further model including both variables as moderators. When controlling for publication year, the effects of NFC (*r* = 0.33) and TIE (*r* = 0.42), as well as the difference between them, became even larger. Despite their high conceptual overlap, NFC and TIE thus seem to differ in the strength of their associations with Gf and Gc: TIE relates more strongly to acquired knowledge whereas NFC is more strongly associated with abstract reasoning ability.

One explanation for this finding, at least in case of Gc, may be the slight conceptual differences between TIE and NFC mentioned above. TIE has been found to comprise at least three dimensions, one of which reflects the tendency to engage in reading (Ackerman and Goff 1994). While the preference for and engagement in effortful thought characteristic of NFC should also lead such individuals to more strongly engage in reading, TIE in that sense seems to capture somewhat broader intellectual lifestyle preferences. Although there are many opportunities for information acquisition today, reading is arguably still a main source of knowledge and certainly was during most of the period covered by the primary studies in our data. This may help explain the stronger associations of TIE with Gc.

However, it should also be noted that the data underlying the TIE effects are considerably smaller, with only 11 effects on TIE and Gf (13 effects in the analysis on Gc). Accordingly, confidence intervals suggest that the TIE estimates are less precise than those for NFC. Moreover, while there is a large diversity in Gf measures in studies on NFC, the effects for TIE were derived from relatively homogeneous sources, many based on aggregated measures comprising multiple single tests (see Supplement E, Tables S1–S6). In case of Gc, these yielded particularly high correlations with TIE (e.g., *r* = 0.37, Rolfhus and Ackerman 1999; *r* = 0.42, Beier and Ackerman 2001), arguably strongly increasing the pooled effect. Thus, the stronger association of TIE with Gc may also partly reflect these methodological differences. This explanation, however, cannot account for the lower TIE-Gf association, as no comparable differences in measurement were evident here.

A second noteworthy result of the moderator analyses was that publication year turned out to be negatively associated with effect size. The only significant result came from the analysis on NFC/TIE and Gc, yet the direction of this effect was relatively consistent across analyses. One explanation for this finding may be increasing pressure from the scientific community to publish not only large and significant effects.

Although no significant differences were found between task characteristics, deductive reasoning showed a considerably weaker association with NFC/TIE than inductive, spatial, or mixed reasoning. However, this result was based on only five effects in the deductive reasoning group, three of which came from a single test (Employee Aptitude Survey; Ruch and Ruch 1980) used across different samples. The finding might hence be methodologically influenced by the characteristics of this particular test. We also examined whether the type of material used (e.g., verbal, figural) moderated the effect by determining the content of the reasoning operation. Differences were again not significant, yet tasks using verbal material showed lower associations than those using figural or mixed material. Notably, five of the eight effects in the verbal reasoning group overlapped with those of the deductive reasoning group, again suggesting that the effect might be driven by these specific tasks. Moreover, since NFC/TIE had the strongest association with verbal knowledge in the present study, these results should be interpreted with caution.

For NFC/TIE and Gc, we also examined differences between aspects of Gc and found the strongest association with general knowledge (*r* = 0.29). Conceptually, this may reflect that individuals high in NFC/TIE tend to be broadly curious about their environment and motivated to understand how the world works. This may lead them to acquire diverse knowledge rather focusing just on specific domains such as verbal knowledge. Supporting this, both NFC and TIE scales have been found to be more strongly associated with broad total knowledge composites than with most specific knowledge domains (e.g., science, humanities) (Ackerman et al. 2001; Von Stumm 2013). However, a methodological explanation may also account for this, as the general knowledge group included several relatively large effects with TIE (e.g., Furnham et al. 2009, *r* = 0.50; Ackerman et al. 2001, *r* = 0.42), which might have contributed strongly to this summary effect.

As the data did not allow for a classification of effects into groups representing task characteristics in the model on general intelligence and shifting, we examined such moderators only in the models on WM and inhibition. Although no significant moderation was found, there was a tendency for interference control (*r* = 0.07) to be more strongly related to NFC than response inhibition (*r* = 0.01). Although group sizes were small (9 and 11 effects, respectively), this is insofar consistent with the conceptualization of NFC, as extended cognitive challenges may less often require suppressing an immediate (especially a motor-) response. Instead, what should be more relevant is the ability to ignore irrelevant information, as reflected by the results. It should be noted, however, that the correlation with interference control did also not reach significance in our analysis (*p* = .054). Regarding the distinction between WM capacity and the updating function, no significant differences were observed. The effect of NFC/TIE with tasks measuring WM capacity was about twice as large (*r* = 0.15) as that with tasks primarily targeting the updating function (*r* = 0.08), but only the former was significant (*p* < .001). A conceptual explanation for this might be that in deep and complex thinking, the ability to quickly update information may be less relevant than the ability to continuously keep in mind what is required to grasp an idea and acquire knowledge.

### 4.3. Longitudinal Relationships Between NFC/TIE and Intelligence

The present meta-analysis focused on the substantial cross-sectional literature on the relationship of NFC/TIE and cognitive ability as well as moderator- and regression analyses to explain variation in effects. However, to further test explanations of these relationships proposed by investment theory (Ackerman 1996) and the OFCI model (Ziegler et al. 2012), longitudinal designs are indispensable. Over the past few years, a small but growing body of longitudinal work has begun to apply such designs (e.g., Hülür et al. 2018; Bergold et al. 2023; Bergold and Steinmayr 2024; Hufer-Thamm et al. 2025; Scherrer et al. 2024), yet the evidence currently remains mixed. For instance, Bergold et al. (2023) found that adolescents with higher Gf showed stronger growth in the hope for success facet of achievement motivation, which reflects desire for mastering achievement-related challenges. However, neither Gf nor Gc predicted changes in NFC (Bergold et al. 2023). Moreover, Bergold and Steinmayr (2024) reported mathematical ability and Gc to predict small to moderate increases in NFC among elementary school children. Scherrer et al. (2024) extended these findings by analyzing reciprocal effects in a latent change score model over one year. Here, when controlling for initial Gc, initial Gf significantly predicted changes in NFC to a moderate degree.

Although effects differ across studies and not all became significant, this provides some support for the environmental success hypothesis. Concerning the environmental enrichment hypothesis of the OFCI model, the current empirical evidence appears to be weaker. Scherrer et al. (2024) indeed found initial NFC to significantly predict gains in Gf over one year and, controlling for Gf and Gc, initial NFC positively but not significantly predicted change in Gc to a moderate degree. In contrast, Hülür et al. (2018) examined adolescents over two years and found growth in Gf not to be predicted by TIE and only a small, non-significant prediction of Gc. Consistent with that, Bergold and Steinmayr (2024) found a significant positive effect of NFC on change in reading comprehension but only a small and non-significant effect on change in Gc.

A limitation for a quantitative synthesis based on these studies is their relatively small scope and their variability in conceptual focus. Some investigations test unidirectional predictions—such as whether TIE predicts subsequent changes in Gf (e.g., Hülür et al. 2018)—while others examine the reverse direction or model reciprocal relationships between cognitive motivation and ability (e.g., Bergold and Steinmayr 2024; Scherrer et al. 2024). Moreover, the existing studies differ in how they operationalize investment traits. For instance, Hülür et al. (2018) used manifest composite scores to reflect TIE, while Bergold and Steinmayr (2024) as well as Hufer-Thamm et al. (2025) used single-indicator latent variables, and Scherrer et al. (2024) modelled NFC as a multi-indicator latent construct. Similarly, Bergold et al. (2023) modelled NFC based on 10 selected items from the-16 item German NFC scale (Bless et al. 1994). Measurement models thus range from manifest composites to latent variables which account for measurement error, albeit with different implementations.

Finally, studies differ in how relationships over time are modelled, in the number of measurement occasions, and interval length. For example, Bergold et al. (2023) followed participants across four measurement occasions over three years and estimated second-order latent growth models to predict interindividual differences in changes in investment traits. In contrast, all other studies relied on two-wave designs, yet vary considerably in time span and the specific analysis (e.g., ranging from latent change score models to observed bivariate longitudinal correlations).

The methodological choices in such demanding study designs are of course constrained by the available resources. However, given that the currently available data come from German educational contexts, participants in school age, and occasionally from academically selective tracks (Scherrer et al. 2024), generalizability is considerably limited. Moreover, to further advance the field, the inclusion of more than just two measurement occasions would allow for a more reliable examination of individual developmental trajectories as well as theoretically implied predictors. Beyond the inclusion of potentially relevant moderators (e.g., self-determination and learning opportunities), this would also allow for the testing of mediating pathways in educational contexts (e.g., academic success mediating the effect of Gf on NFC).

Overall, the scarcity of overall longitudinal data, combined with the diversity in methodology and focus, currently makes it difficult to integrate this promising primary study data in meta-analytic models. Using similar study designs and comparable statistical models as a common methodological basis would allow for a reliable synthesis of not only cross-sectional but also longitudinal findings.

### 4.4. Limitations

The findings of the present study are not without limitations. First, as evident from the PRISMA chart, we identified potentially relevant records for which the full text could not be accessed, relevant data was not obtainable, or the methodological criteria were not met. Although the large number of included effects in the analyses on Gf, Gc, and WM mitigates this issue to some extent, it remains a limitation for the analyses on general intelligence, inhibition, and especially shifting, where the empirical base was smaller. Similarly, we obtained data from several WM tasks in which the dependent variable was not clearly described. These effects had to be excluded because it was unclear whether the underlying measure or calculated outcome was appropriate. This was less problematic in the analyses on intelligence, as the reported outcomes usually reflect accuracy (e.g., number of correct items). It is hence highly plausible that this was also the case when information was missing.

A further limitation concerns the coding process. The two screeners and coders worked independently during the title and abstract as well as full-text screening, yet due to the large amount of data, this was changed for coding. Here, each coder merely validated and, if necessary, corrected the other’s work during coding. Although this validation was carefully and transparently carried out, a fully independent coding procedure would have been preferable.

Further addressing the last point of the previous section concerning the role of time-constraints in cognitive challenges, only in a few cases did the authors provide detailed information on whether time for test completion was limited. Consequently, too little information was available to examine temporal constraints as a potential moderator. Yet even if information on the exact allotted time had been available, this alone would not have been a well-operationalized predictor, as intelligence tasks are quite heterogenous in both the number of items and item difficulty. Considering that high-NFC/TIE individuals engage longer and more thoroughly with a task (e.g., Fleischhauer et al. 2010), this made it impossible to analyze this arguably very informative moderator of our effects.

As often noted, self-report scales can be strongly influenced by self-presentation effects (e.g., Krumpal 2013), limiting the extent to which scores on such scales reflect true construct expressions. A further methodological problem concerning executive functions is that these reflect relatively narrow abilities, usually measured with tests of low bandwidth. Pairing comparatively broad constructs like NFC/TIE with executive functions might therefore result in a lack of Brunswik symmetry (Wittmann and Süß 1999); that is, a mismatch between the breadth of the examined constructs and hence the corresponding tests (Ackerman et al. 2005). One remedy might be to measure a given function using multiple tasks. The current literature, however, seems to rely mostly on low-bandwidth single measures, which prevents more precise effect size estimations.

A further limitation associated with the primary study data is that most of the effects we found were based on the raw score of a task, such as the number of correctly solved items. Because the primary data were available in only very few cases, it was not possible to obtain consistently age-normed effects, leading us to include both standardized and unstandardized effects in the meta-analyses. As with the prior limitation, such differences cannot be easily analyzed in moderator analyses, as the presence or absence of standardization and the age distribution of the sample would need to be taken into account.

Concerning age as a potential moderator, the available data did not allow us to allocate samples to non-overlapping age groups, as these often show considerable variation. To avoid disregarding this potentially informative moderator, we operationalized age differences using the mean age of the sample. Although this procedure is common in meta-analyses (e.g., Buecker et al. 2020; Luo et al. 2023), it has been argued (e.g., Schwarzer et al. 2015, Harrer et al. 2022) that conclusions from moderator analyses based solely on mean age can be misleading, since effects may be driven by only a small subgroup (e.g., younger adults in a sample of mostly older adults). As mean age is therefore a relatively imprecise indicator of the age characteristics of a sample, these results should be interpreted with caution. The same applies to the percentage of female (or male) participants as an indicator of gender. As Craig Aulisi et al. (2023) argue, primary studies often show very similar male-to-female ratios, leaving little variation across samples. This, in turn, makes it difficult to detect gender differences in meta-analytic moderator analyses, even if they exist. A clearer allocation into exclusively male or exclusively female samples would have allowed us to examine this moderator without this limitation. However, as shown by the descriptive statistics, our data did not permit this.

A more general limitation concerns the heterogeneity diagnostics. The sample sizes of the individual effects were relatively substantial in the analyses on Gf, Gc, general intelligence, and WM, but smaller in the analyses on inhibition and shifting. This poses a challenge for the interpretation of these results, as studies (e.g., Li et al. 2015) have shown that both the *Q*-test and the *I*^2^ statistic tend to increase with sample size for the individual effects, even in the absence of heterogeneity. In the analyses with large sample sizes for the primary effects, the significant results of the *Q*-test and *I*^2^ may thus also be partly methodologically driven. A further issue with the *I*^2^ statistic is that it yields biased results when the number of studies in a meta-analysis is small, which is particularly problematic for the analysis on shifting. Nonetheless, it suggests that at least some of our analyses do indeed reflect substantial heterogeneity is the size of the prediction intervals, indicating the range in which a new effect would fall if selected at random from the population (Borenstein et al. 2009). Given these uncertainties in the diagnostic tools, we conducted moderator analyses for all models.

In the present analysis, we chose a multi-level approach to capture the structure of the data. The often-used alternative of calculating the mean of dependent effects within a cluster (Borenstein et al. 2009) and analyzing this single effect would, in our case, have substantially reduced the number of effects (see descriptives). This is because there were not only multiple effects clustered within a sample but also samples within publications and, in some analyses, multiple effects within a single task. Rather than completely eliminating this nested structure, we opted for multi-level models combined with RVE to retain as many effects as possible. While we regard multi-level modelling as preferable to alternatives, particularly when combined with RVE, it also entails limitations regarding the applicability of some commonly used meta-analytic methods, such as the trim-and-fill analysis. However, the trim-and-fill analysis has also been shown to produce invalid results when heterogeneity is high (Terrin et al. 2003). As this was the case in some of our analyses, its findings would have had to be interpreted with caution anyway.

## 5. Conclusions

NFC and TIE are two of the most well-known and closely linked constructs representing inter-individual differences in cognitive motivation. With the present multi-level meta-analysis, we aimed to clarify their associations with Gf, Gc, and general intelligence, as well as WM capacity, updating, shifting, and inhibition. In doing so, we examined potential factors underlying differences between effects, whether NFC and TIE are differently related to cognitive functions, and finally whether associations with intelligence generalize to more basic executive functions. Results provide strong evidence for small-to-moderate associations of NFC/TIE with Gf, Gc, general intelligence, and WM capacity, at best weak associations with WM updating, and no associations with shifting and inhibition. The associations of NFC/TIE with intelligence thus do not seem to generalize to core executive functions. However, while the analyses on Gf (*N* = 25,367, *e* = 76), Gc (*N* = 14,651, *e* = 65), general intelligence (*N* = 8479, *e* = 24), and WM capacity (*N* = 5921, *e* = 40) were based on a substantial number of effects and participants, the empirical base was smaller for updating (*N* = 1084, *e* = 10), inhibition (*N* = 2895, *e* = 21), and shifting (*N* = 1727, *e* = 13). Importantly, we found NFC to be significantly more strongly associated with Gf than TIE, whereas TIE related more strongly to Gc than NFC. Although they are conceptually and empirically highly associated, NFC and TIE thus seem to differ in their relation to Gf and Gc. Limitations of the present study include small subgroup sizes for some moderators as well as common methodological decisions in the measurement of cognitive ability, such as the frequent use of unstandardized test scores. This highlights the importance of further examining the relationship of NFC/TIE with these functions in high-quality primary studies.

## Figures and Tables

**Figure 1:**
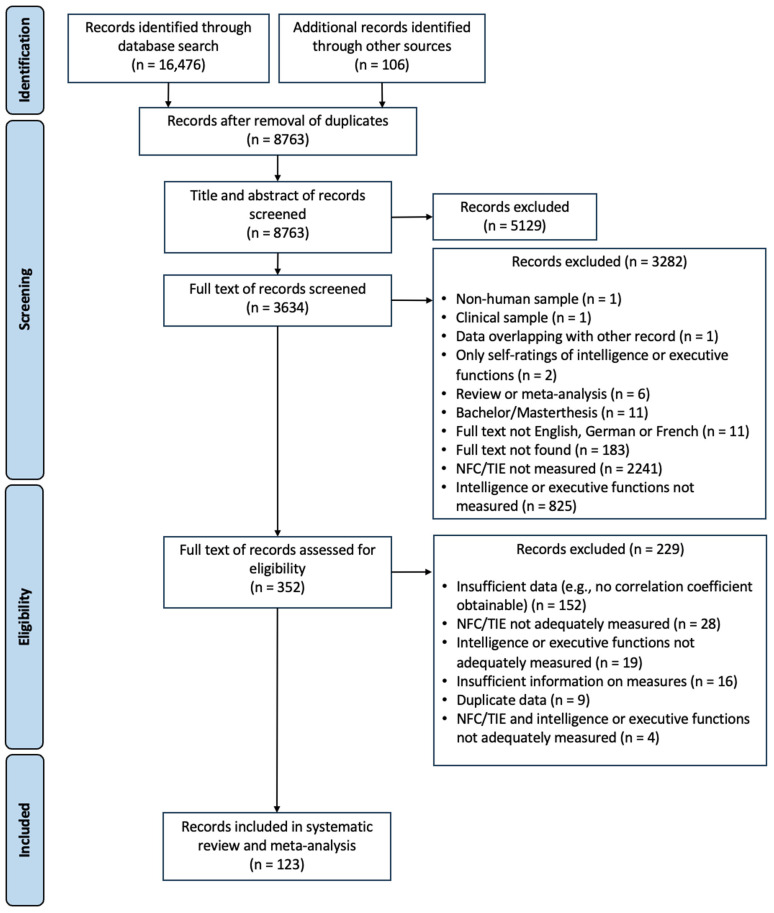
PRISMA Diagram.

**Figure 2:**
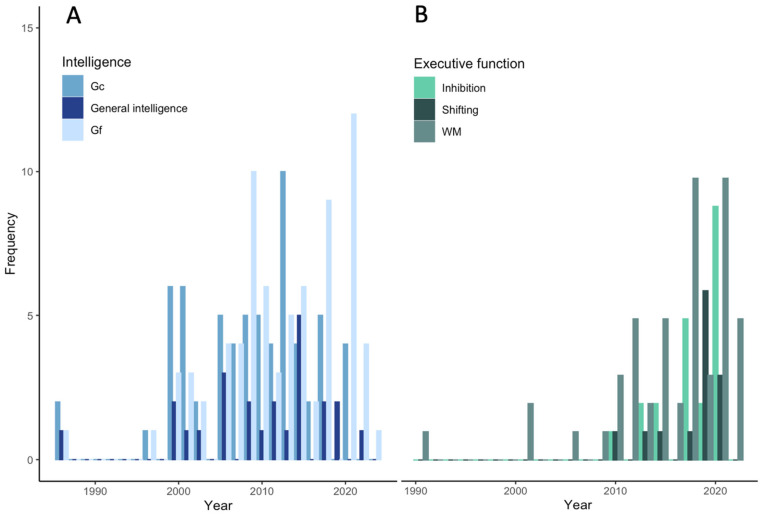
Histogram on Publication Year for Effects of NFC/TIE with Intelligence and Executive Functions. Gf = fluid intelligence; Gc = crystallized intelligence; WM = working memory; (**A**): effects in meta-analyses on NFC/TIE and intelligence; (**B**): effects in meta-analyses on NFC/TIE and executive functions.

**Table 1:** Overall Association of NFC/TIE with Intelligence and Executive Functions.

Cognitive Function		*k*	*s*	*t*	*e*	*N*	*r*	95% CI	*p*	τ_level 5_	τ_level 4_	τ_level 3_	τ_level 2_	*I* ^2^_level 5_	*I* ^2^_level 4_	*I* ^2^_level 3_	*I* ^2^_level 2_	*Q*(df)
*Intelligence*																		
	Gf	61	69	76	76	25,367	0.18	[0.15, 0.20]	<.001	0.000	0.094	0.000		0.00	72.89	0.00		*Q*(75) = 232.81 ***
	Gc	51	56	65	65	14,651	0.26	[0.23, 0.29]	<.001	0.086	0.000	0.030		58.19	0.00	6.70		*Q*(64) = 179.71 ***
	General intelligence	24	24	24	24	8479	0.23	[0.18, 0.28]	<.001	0.108 ***				78.10				*Q*(23) = 78.99 ***
*Executive functions*																		
	WM	36	41	45	50	7005	0.14	[0.10, 0.18]	<.001	0.058	0.078	0.000	0.000	19.83	36.13	0.00	0.00	*Q*(49) = 93.16 ***
	Inhibition	12	13	19	21	2895	0.04	[−0.01, 0.09]	.077	0.000	0.018	0.040	0.000	0.00	3.56	16.91	0.00	*Q*(20) = 27.92
	Shifting	8	9	10	13	1727	0.01	[−0.05, 0.07]	.642	0.000	0.019	0.000	0.000	0.00	4.42	0.00	0.00	*Q*(12) = 9.95

Note. NFC = need for cognition; TIE = typical intellectual engagement; *k* = number of publications; *s* = number of samples; *t* = number of single tasks; *e* = number of effects; *N* = number of participants; *r* = meta-analytic Pearson’s r; CI = confidence interval; τ_level 2_ = within-task variance; τ_level 3_ = between-task variance; τ_level 4_ = between-sample variance; τ_level 5_ *=* between-publication variance; *I*^2^_level 2_ = % of total variance attributable to within-task differences; *I*^2^_level 3_ = % of total variance attributable to within-sample differences; *I*^2^_level 4_ = % of total variance attributable to between-sample differences; *I*^2^_level 5_ = % of total variance attributable to between-publication differences; *Q* = *Q*-test for heterogeneity; Gf = fluid intelligence; Gc = crystallized intelligence; WM = working memory; *** *p* < .001.

**Table 2:** Moderator Effects on the Association of NFC/TIE with Gf.

Moderator		Comparison	*r*	95% CI	*p*	*b*_1_	β_1_	95% CI	*p*	*F*(df1, df2)	*Q*(df)
Publication year			0.16	[0.04, 0.27]	.010	0.001	0.01	[−0.03, 0.04]	.699		*Q*(74) = 232.24 ***
Publication											*Q*(74) = 231.81 ***
	Journal	Dissertation	0.17	[0.14, 0.20]	<.001					*F*(1, 8.58) = 0.73	
	Dissertation		0.21	[0.10, 0.32]	.003						
Mean age			0.14	[0.08, 0.19]	<.001	0.002	0.02	[−0.01, 0.06]	.158		*Q*(60) = 192.95 ***
	Red. model		0.17	[0.14, 0.20]	<.001						*Q*(61) = 199.64 ***
% female			0.16	[0.05, 0.27]	.009	0.000	0.00	[−0.03, 0.03]	.919		*Q*(69) = 208.73 ***
	Red. model		0.17	[0.14, 0.20]	<.001						*Q*(70) = 210.80 ***
Risk of bias			0.11	[0.02, 0.18]	.011	0.027	0.03	[0.00, 0.06]	.070		*Q*(74) = 232.11 ***
College											*Q*(74) = 215.20 ***
	No	Yes	0.19	[0.14, 0.24]	<.001					*F*(1, 47.10) = 0.97	
	Yes		0.16	[0.13; .20]	<.001						
Controlled											*Q*(69) = 194.85 ***
	No	Yes	0.18	[0.12, 0.24]	<.001					*F*(1, 24.70) = 0.03	
	Yes		0.17	[0.14, 0.21]	<.001						
	Red. model		0.17	[0.15, 0.20]	<.001						*Q*(70) = 198.02 ***
Simultaneous											*Q*(70) = 194.68 ***
	No	Yes	0.17	[0.09, 0.25]	<.001					*F*(1, 21.50) = 0.05	
	Yes		0.18	[0.15, 0.21]	<.001						
	Red. model		0.17	[0.14, 0.20]	<.001						*Q*(71) = 196.29 ***
Aspect											*Q*(72) = 214.35 ***
	Inductive		0.19	[0.15, 0.23]	<.001						
		Deductive								*F*(1, 6.57) = 5.39	
		Spatial								*F*(1, 10.72) = 0.80	
		Mixed								*F*(1, 18.88) = 0.00	
	Deductive		0.10	[0.02, 0.19]	.024						
		Spatial								*F*(1, 9.42) = 1.82	
		Mixed								*F*(1, 9.77) = 3.63	
	Spatial		0.16	[0.10, 0.23]	<.001						
		Mixed								*F*(1, 18.70) = 0.45	
	Mixed		0.19	[0.12, 0.26]	<.001						
Content											*Q*(73) = 208.74 ***
	Figural		0.19	[0.15, 0.22]	<.001						
		Verbal								*F*(1, 4.65) = 11.15	
		Mixed								*F*(1, 10.02) = 0.00	
	Verbal		0.11	[0.05, 0.16]	.005						
		Mixed								*F*(1, 4.29) = 8.96	
	Mixed		0.18	[0.13, 0.24]	<.001						
NFC/TIE scale											*Q*(74) = 232.68 ***
	NFC	TIE	0.19	[0.16, 0.22]	<.001					*F*(1, 12.10) = 5.04 *	
	TIE		0.12	[0.05, 0.18]	.003						

Note. NFC = need for cognition; TIE = typical intellectual engagement; Gf = fluid intelligence; *r* = intercept in regression analyses with continuous predictors and meta-analytic correlation coefficient in different groups in case of categorical moderators; *b*_1_ = unstandardized regression coefficient; β_1_ = standardized regression coefficient; CI = confidence interval; *F* = *F*-test; *Q* = *Q*-test for residual heterogeneity; College = exclusively college sample; Controlled = controlled measurement; Content = content of reasoning operation; Simultaneous = simultaneous measurement; Red. model = model without moderators with missing cases removed; *** *p* < .001; * *p* < .05.

**Table 3:** Moderator Effects on the Association of NFC/TIE with Gc.

Moderator		Comparison	*r*	95% CI	*p*	*b*_1_	β_1_	95% CI	*p*	*F*(df1, df2)	*Q*(df)
Publication year			0.38	[0.28, 0.48]	<.001	−0.006	−0.04	[−0.08, −0.01]	.010		*Q*(63) = 153.39 ***
Mean age			0.30	[0.24, 0.35]	<.001	−0.002	−0.03	[−0.07, 0.01]	.113		*Q*(55) = 161.84 ***
	Red. model		0.26	[0.22, 0.30]	<.001						*Q*(56) = 164.87 ***
% female			0.27	[0.10, 0.43]	.004	0.000	0.00	[−0.04, 0.04]	.860		*Q*(59) = 175.57 ***
	Red. model		0.26	[0.23, 0.29]	<.001						*Q*(60) = 175.58 ***
Risk of bias			0.29	[0.18, 0.39]	<.001	−0.010	−0.01	[−0.05, 0.03]	.557		*Q*(63) = 174.00 ***
College											*Q*(63) = 178.64 ***
	No	Yes	0.25	[0.20, 0.30]	<.001					*F*(1, 24.90) = 0.29	
	Yes		0.27	[0.23, 0.31]	<.001						
Controlled											*Q*(63) = 178.61 ***
	No	Yes	0.27	[0.19, 0.34]	<.001					*F*(1, 19.80) = 0.13	
	Yes		0.26	[0.22, 0.30]	<.001						
	Red. model		0.26	[0.23, 0.30]	<.001						*Q*(64) = 179.71 ***
Simultaneous											*Q*(57) = 161.57 ***
	No	Yes	0.24	[0.16, 0.30]	<.001					*F*(1, 24.20) = 0.45	
	Yes		0.26	[0.22, 0.30]	<.001						
	Red. model		0.25	[0.22, 0.29]	<.001						*Q*(58) = 166.35 ***
Aspect											*Q*(61) = 170.75 ***
	Gen. know.		0.29	[0.23, 0.35]	<.001						
		Verb. know.								*F*(1, 18.70) = 1.46	
		Verb. know. +								*F*(1, 8.54) = 1.10	
		reasoning									
		Mixed								*F*(1, 10.50) = 0.67	
	Verb. know.		0.25	[0.21, 0.29]	<.001						
		Verb. know. +								*F*(1, 7.57) = 0.05	
		reasoning									
		Mixed								*F*(1, 11.92) = 0.10	
	Verb. know.		0.24	[0.14, 0.34]	.001						
	+reasoning	Mixed								*F*(1, 9.89) = 0.17	
	Mixed		0.26	[0.20, 0.34]	<.001						
NFC/TIE scale											*Q*(63) = 144.89 ***
	NFC	TIE	0.24	[0.20, 0.27]	<.001					*F*(1, 13.10) = 10.70 **	
	TIE		0.35	[0.28, 0.42]	<.001						

Note. NFC = need for cognition; TIE = typical intellectual engagement; Gc = crystallized intelligence; *r* = intercept in regression analyses with continuous predictors and meta-analytic correlation coefficient in different groups in case of categorical moderators; *b*_1_ = unstandardized regression coefficient; β_1_ = standardized regression coefficient; CI = confidence interval; *F* = *F*-test; *Q* = *Q*-test for residual heterogeneity; College = exclusively college sample; Controlled = controlled measurement; Simultaneous = simultaneous measurement; Red. model = model without moderators with missing cases removed; Gen. know. = general knowledge; Verb. know = verbal knowledge; *** *p* < .001; ** *p* < .01.

**Table 4:** Moderator Effects on the Association of NFC/TIE with General Intelligence.

Moderator		Comparison	*r*	95% CI	*p*	*b*_1_	β_1_	95% CI	*p*	*F*(df1, df2)	*Q*(df)
Publication year			0.30	[0.12, 0.48]	.002	−0.003	−0.03	[−0.08, 0.03]	.390		*Q*(22) = 75.48 ***
Mean age			0.13	[−0.10, 0.35]	.270	0.006	0.03	[−0.03, 0.09]	.323		*Q*(19) = 71.68 ***
	Red. model		0.23	[0.17, 0.30]	<.001						*Q*(20) = 78.32 ***
% female			0.29	[−0.04, 0.61]	.080	−0.001	−0.01	[−0.08, 0.06]	.758		*Q*(17) = 77.26 ***
	Red. model		0.24	[0.17, 0.31]	<.001						*Q*(18) = 77.38 ***
Risk of bias			0.24	[0.11, 0.36]	.001	−0.002	0.00	[−0.06, 0.06]	.941		*Q*(22) = 78.67 ***
College											*Q*(22) = 76.02 ***
	No	Yes	0.21	[0.13, 0.29]	<.001					*F*(1, 22) = 0.43	
	Yes		0.25	[0.17, 0.32]	<.001						
Controlled											*Q*(20) = 78.75 ***
	No	Yes	0.21	[0.06, 0.34]	.006					*F*(1, 20) = 0.24	
	Yes		0.24	[0.17, 0.31]	<.001						
	Red. model		0.24	[0.18, 0.29]	<.001						*Q*(21) = 78.78 ***
Simultaneous											*Q*(20) = 66.85 ***
	No	Yes	0.24	[0.14, 0.34]	<.001					*F*(1, 20) = 0.00	
	Yes		0.24	[0.16, 0.32]	<.001						
	Red. model		0.24	[0.18, 0.30]	<.001						*Q*(21) = 67.77 ***
NFC/TIE scale											*Q*(22) = 76.30 ***
	NFC	TIE	0.22	[0.16, 0.29]	<.001					*F*(1, 22) = 0.32	
	TIE		0.26	[0.15, 0.36]	<.001						

Note. NFC = need for cognition; TIE = typical intellectual engagement; *r* = intercept in regression analyses with continuous predictors and meta-analytic correlation coefficient in different groups in case of categorical moderators; *b*_1_ = unstandardized regression coefficient; β_1_ = standardized regression coefficient; CI = confidence interval; *F* = *F*-test; *Q* = *Q*-test for residual heterogeneity; College = exclusively college sample; Controlled = controlled measurement; Simultaneous = simultaneous measurement; Red. model = model without moderators with missing cases removed; *** *p* < .001.

**Table 5:** Moderator Effects on the Association of NFC/TIE with WM.

Moderator		Comparison	*r*	95% CI	*p*	*b*_1_	β_1_	95% CI	*p*	*F*(df1, df2)	*Q*(df)
Publication year			0.30	[0.09, 0.51]	.015	−0.007	−0.05	[−0.10, 0.01]	.099		*Q*(48) = 82.75 ***
Publication											*Q*(48) = 90.90 ***
	Journal	Dissertation	0.13	[0.08, 0.18]	<.001					*F*(1, 5.29) = 1.65	
	Dissertation		0.19	[0.07, 0.31]	.012						
Mean age			0.11	[0.02, 0.20]	.016	0.001	0.01	[−0.08, 0.10]	.752		*Q*(39) = 81.22 ***
	Red. model		0.12	[0.07, 0.18]	<.001						*Q*(40) = 81.33 ***
% female			0.04	[−0.16, 0.24]	.668	0.002	0.03	[−0.03, 0.09]	.363		*Q*(41) = 81.99 ***
	Red. model		0.13	[0.08, 0.18]	<.001						*Q*(42) = 84.10 ***
Risk of bias			0.23	[0.06, 0.38]	.011	−0.028	−0.03	[−0.06, 0.01]	.175		*Q*(48) = 89.65 ***
College											*Q*(48) = 92.73 ***
	No	Yes	0.14	[0.07, 0.21]	<.001					*F*(1, 29.30) = 0.01	
	Yes		0.14	[0.07, 0.20]	<.001						
Controlled											*Q*(46) = 91.71 ***
	No	Yes	0.12	[0.00, 0.24]	.056					*F*(1, 6.49) = 0.31	
	Yes		0.15	[0.10, 0.20]	<.001						
	Red. model		0.14	[0.10, 0.19]	<.001						*Q*(47) = 91.71 ***
Simultaneous											*Q*(39) = 83.80 ***
	No	Yes	0.10	[−0.10, 0.30]	.217					*F*(1, 4.48) = 0.46	
	Yes		0.15	[0.10, 0.20]	<.001						
	Red. model		0.14	[0.09, 0.19]	<.001						*Q*(40) = 85.37 ***
Function											*Q*(48) = 92.90 ***
	Updating	Capacity	0.08	[−0.03, 0.18]	.111					*F*(1, 6.81) = 2.91	
	Capacity		0.15	[0.10, 0.20]	<.001						

Note. NFC = need for cognition; TIE = typical intellectual engagement; WM = working memory; *r* = intercept in regression analyses with continuous predictors and meta-analytic correlation coefficient in different groups in case of categorical moderators; *b*_1_ = unstandardized regression coefficient; β_1_ = standardized regression coefficient; CI = confidence interval; *F* = *F*-test; *Q* = *Q*-test for residual heterogeneity; College = exclusively college sample; Controlled = controlled measurement; Simultaneous = simultaneous measurement; Red. model = model without moderators with missing cases removed; *** *p* < .001.

**Table 6:** Moderator Effects on the Association of NFC with Inhibition.

Moderator		Comparison	*r*	95% CI	*p*	*b*_1_	β_1_	95% CI	*p*	*F*(df1, df2)	*Q*(df)
Publication year			0.05	[−0.11, 0.21]	.363	−0.001	0.00	[−0.06, 0.05]	.795		*Q*(19) = 27.87
Mean age			0.01	[−0.06, 0.08]	.640	0.005	0.02	[−0.10, 0.14]	.596		*Q*(17) = 22.66
	Red. model		0.03	[−0.03, 0.09]	.208						*Q*(18) = 23.03
% female			−0.17	[−0.65, 0.31]	.422	0.003	0.03	[−0.05, 0.12]	.360		*Q*(17) = 22.60
	Red. model		0.03	[−0.03, 0.09]	.208						*Q*(18) = 23.03
Risk of bias			0.10	[−0.11, 0.31]	.218	−0.021	−0.02	[−0.10, 0.06]	.428		*Q*(19) = 26.60
College											*Q*(19) = 27.85
	No	Yes	0.06	[−0.08, 0.20]	.347					*F*(1, 7.67) = 0.23	
	Yes		0.03	[−0.04, 0.10]	.217						
Simultaneous											*Q*(17) = 26.03
	No	Yes	0.03	[−0.28, 0.32]	.614					*F*(1, 2.57) = 0.33	
	Yes		0.06	[−0.01, 0.13]	.098						
	Red. model		0.05	[−0.01, 0.11]	.087						*Q*(18) = 26.03
Function											*Q*(18) = 23.71
	Interf. cont.	Resp. inhib.	0.07	[0.00, 0.15]	.054					*F*(1, 3.36) = 5.13	
	Resp. inhib.		0.01	[−0.04, 0.06]	.465						
	Red. model		0.05	[0.00, 0.10]	.041						*Q*(19) = 25.72

Note. NFC = need for cognition; *r* = intercept in regression analyses with continuous predictors and meta-analytic correlation coefficient in different groups in case of categorical moderators; *b*_1_ = unstandardized regression coefficient; β_1_ = standardized regression coefficient; CI = confidence interval; *F* = *F*-test; *Q* = *Q*-test for residual heterogeneity; College = exclusively college sample; Simultaneous = simultaneous measurement; Interf. cont. = interference control; Resp. inhib. = response inhibition; Red. model = model without moderators with missing cases removed.

**Table 7:** Moderator Effects on the Association of NFC with Shifting.

Moderator		Comparison	*r*	95% CI	*p*	*b*_1_	β_1_	95% CI	*p*	*F*(df1, df2)	*Q*(df)
Publication year			0.06	[−0.14, 0.25]	.228	−0.007	−0.02	[−0.10, 0.05]	.325		*Q*(11) = 8.59
Mean age			−0.06	[−0.12, 0.00]	.059	0.005	0.05	[−0.01, 0.10]	.068		*Q*(10) = 5.00
	Red. model		0.00	[−0.07, 0.07]	.957						*Q*(11) = 9.30
% female			0.04	[−0.22, 0.30]	.671	−0.001	−0.01	[−0.07, 0.04]	.424		*Q*(9) = 6.76
	Red. model		−0.02	[−0.09, 0.05]	.473						*Q*(10) = 6.96
Risk of bias			−0.03	[−0.24, 0.18]	.614	0.013	0.01	[−0.05, 0.08]	.482		*Q*(11) = 9.66
College											*Q*(11) = 7.67
	No	Yes	0.06	[−0.04, 0.16]	.138					*F*(1, 4.67) = 3.28	
	Yes		−0.01	[−0.12, 0.09]	.668						

Note. NFC = need for cognition; *r* = intercept in regression analyses with continuous predictors and meta-analytic correlation coefficient in different groups in case of categorical moderators; *b*_1_ = unstandardized regression coefficient; β_1_ = standardized regression coefficient; CI = confidence interval; *F* = *F*-test; *Q* = *Q*-test for residual heterogeneity; College = exclusively college sample; Red. model = model without moderators with missing cases removed.

## Data Availability

Data and analysis code are available at https://osf.io/7n58e/overview.

## Notes

1 The direction of the effect reflects lower theta power in high-NFC individuals in simple compared to complex tasks.

2 We originally intended to focus only on Gf and Gc. However, the inspection of the literature following preregistration revealed several studies using broad measures of intelligence that can be considered proxies for general intelligence. Although the scope of this analysis is smaller than that for Gf and Gc, we decided to include these effects in a separate analysis. Similarly to the processing of the literature on general intelligence in Anglim et al. (2022), the included measures had to be sufficiently broad – either targeting at least two ability domains or consisting of a single broad measure with tasks covering several different abilities, such as the Wonderlic Personnel Test (Wonderlic Inc. 1999) or Wechsler Adult Intelligence Scale (WAIS–III; Wechsler 1997). We will refer to this as *general intelligence*, although arguably not every measure found in the literature perfectly aligns with a general g-factor (Spearman 1904).

3 Similarly, we identified many studies examining the associations of NFC/TIE with WM capacity and not specifically updating. Meta-analytic results from these data are also presented below for the interested reader.

4 For simplicity we refer to this moderator as “publication year” in the following.

## Supplementary Materials

The following supporting information can be downloaded at https://osf.io/7n58e/overview (accessed on 26 October 2025): further information on methods and the literature search, screening and coding tools, forest plots, descriptive statistics, information on risk of bias assessment, reliabilities of scales and tests, figures on publication bias assessment, results corrected for attenuation, amendments and annotations to preregistration, and references of scales and tests.

## Abbreviations

The following abbreviations are used in this manuscript:

NFC	need for cognition
TIE	typical intellectual engagement
NFC/TIE	need for cognition/typical intellectual engagement
WM	working memory
Gc	crystallized intelligence
Gf	fluid intelligence
Gen. know.	general knowledge
Verb. know	verbal knowledge
Resp. inhib.	response inhibition
Interf. cont.	interference control
Red. model	model without moderators with missing cases removed
OFCI	Openness-Fluid-Crystallized-Intelligence
PET-PEESE	precision-effect test and precision-effect estimate with standard errors
CHC	Cattell-Horn-Carroll
RVE	robust variance estimation
ROBUST	Risk of Bias Utilized for Surveys Tool
REML	restricted maximum likelihood
CI	confidence interval
PI	prediction interval
SE	standard error
k	number of publications
s	number of studies
t	number of tasks
e	number of effects
DGPs	Deutsche Gesellschaft für Psychologie
